# Probing the ^11^B Quadrupolar and Chemical Shielding Tensors in a Pair of Organoboron Enantiomers

**DOI:** 10.1021/acs.jpca.5c03645

**Published:** 2025-11-11

**Authors:** Shiva Agarwal, Zhongrui Li, Jason Kitchen, Sungsool Wi, John B. Miller

**Affiliations:** † Department of Physics, 4175 Western Michigan University, Kalamazoo, Michigan 49008, United States; ‡ Electron Microbeam Analysis Laboratory, 1259 University of Michigan, Ann Arbor, Michigan 48109, United States; § 189689 National High Magnetic Field Laboratory, Tallahassee, Florida 32310, United States; ∥ Department of Chemistry, Western Michigan University, Kalamazoo, Michigan 49008, United States

## Abstract

Chirality plays a fundamental role in numerous scientific fields, yet the electronic structures of chiral compounds, particularly pairs of enantiomers, remain understudied. In this study, we address this gap by investigating the electronic structure of a chiral pair through single-crystal nuclear magnetic resonance (NMR) spectroscopy, complemented by X-ray diffraction and density functional theory (DFT) calculations. The combination of these techniques allows for the precise determination of the electronic environments of chiral molecules, offering direct insights into the subtle differences between enantiomers. Our results demonstrate the robustness of the capacity for experiment and computation to combine in resolving the NMR interaction tensors of enantiomers. This study advances our understanding of the chiral electronic structures of high-spin nuclei and the effects of chirality in various scientific contexts, although the potential for single-crystal NMR in stereochemical analysis remains a challenge for high-spin nuclei in asymmetric environments.

## Graphical abstract

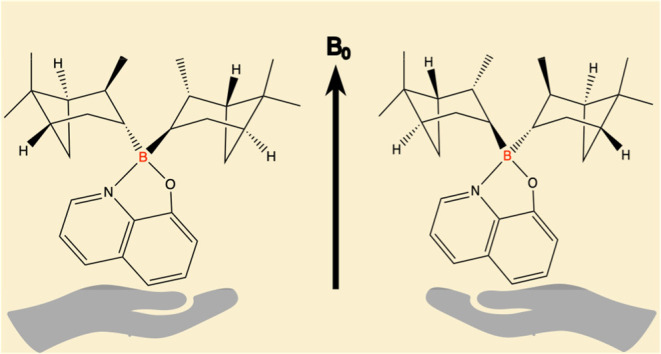

## Introduction

Chirality plays a fundamental role in nature and in our lives. Amino acids—the building blocks of proteins essential to all living organisms—are chiral (except glycine), and this molecular handedness profoundly influences biological processes. For instance, the binding of drug molecules in the human body is highly dependent on their stereochemistry. This insight has driven the rapid development of single-enantiomer drugs.^1^ Beyond pharmacology, chirality is also critical to how our nervous system interprets signals from olfactory receptors and taste buds.^2,3^ Despite its significance, the origin of biological homochirality^4^ remains an open question in science.

One promising avenue of research involves using nuclear magnetic resonance (NMR) spectroscopy to study quadrupolar and chemical shift tensors in chiral compounds. These studies, often supported by density functional theory (DFT) calculations, may have far-reaching implications in fields such as astrobiology, pharmaceuticals, materials science, and food safety.^5–8^ This study investigates these tensors in a pair of enantiomers, offering new insights into the role of chirality in NMR spectroscopy and its broader scientific impact.

A key motivation for this work lies in the theoretical prediction that chemical shielding—particularly the antisymmetric chemical shielding (ACS) components—can differentially influence nuclear interactions in chiral environments, especially under high electric and magnetic fields. This concept is central to the so-called magnetochiral model,^9^ which proposes that such field-induced asymmetries could impact nuclear reactions involving relativistic polarized leptons. One example is the reaction ^14^N + *ν̅*_*e*_ → ^14^C + e^+^, which has been proposed as a potential mechanism contributing to the enantiomeric excess of *L*-amino acids in certain carbonaceous chondrite meteorites^10,11^ by preferential destruction of the *D-*enantiomer.

While NMR spectroscopy is a powerful tool for studying the electronic structure of molecules, its application to chiral systems has been limited. This is primarily because the distinguishing feature of chirality in NMR arises from the antisymmetric component of the chemical shielding tensor (ACS),^12^ which has rarely been directly observed. The chemical shielding tensor has the ability to differentiate between enantiomers when fully considering all the components including the antisymmetric parts.^13–15^ Given that there is no firm theoretical basis for CPMAS to discriminate between enantiomers, the best chance to differentiate them is by studying their ACS tensor components,^16^ as there is no evidence that the symmetric parts of NMR tensors alone can do so. To date, ACS contributions have only been indirectly inferred through relaxation studies in solution-state NMR^17,18^ or, with large uncertainties, from quadrupolar-ACS cross-correlation data in single-crystal ^59^Co NMR.^19^

The limited experimental ACS-component data are primarily a result of the ACS components’ contribution through second-order perturbation effects, which makes their influence on conventional spin-1/2 NMR spectra relatively small. Even for quadrupolar nuclei (*I* > 1/2), measurement of the ACS components requires observing the satellite transitions, which are often weak and sensitive to NMR experimental parameters.^20^

In this work, we have investigated the quadrupolar tensor and the chemical shielding anisotropy (CSA) constituent of the chemical shielding tensor, along with their relative orientations for ^11^B (*I* = 3/2, γ_11B_ = 8.58 × 10^7^ rad T^–1^s^–1^), providing a complete magnetic picture of the ^11^B nuclei in the crystal structure of *B,B,B,B*-8-hydroxyquinoline-*bis*-isopinocampheyl-borane (8-HQ­(ipc)_2_B). Complementary to experimental methods, electronic structure calculations such as density functional theory (DFT) continue to play a vital role in deepening our understanding of shielding tensors and the internal electronic environments of chiral systems.^21^ We have compared the experimental results with computations of the relevant NMR interaction parameters in periodic model systems based on the published crystal structure.^22^ Experimentally, we were ultimately unsuccessful in measuring the ACS tensor components. From the DFT calculations, which included the GIPAW method^23^ as an overlay to compute the NMR and electric field gradient (EFG) tensor, we obtained that ACS tensor information along with the conventional quadrupolar and chemical shift anisotropy tensor components. GIPAW results have been shown to be comparable to experiment for CSA and J-coupling.^17,24^ In the case of the chiral enantiomeric pair, we observed that while the quadrupolar, CSA, and absolute values of the ACS components were identical for both, the signs of the ACS components were reversed, reflecting their inherent handedness.

To carry out this investigation, we sought a relatively sensitive quadrupolar nucleus in a chiral environment. Ultimately, we selected the (+,+) and (-,-) enantiomers of 8-HQ­(ipc)_2_B. This compound is a derivative of a commonly used chiral catalyst, so the enantiomerically pure precursors were commercially available. An important advantage of the 8-HQ­(ipc)_2_B compound is that the hydroxyquinolyl nitrogen is quadrupolar (*I* = 1) and in the boron coordination sphere. Having both nuclei provided the potential opportunity to examine the impact of an antisymmetric environment on nuclei not directly bonded to optically active centers—the aromatic nitrogen is trigonal planar and thus not inherently chiral—as well as any *J-*coupling between the B and N quadrupolar nuclei.

Our attempts to observe the inherently low-sensitivity ^14^N (γ = 1.93 × 10^7^ rad *T* ^–1^ *s* ^–1^) nucleus in this molecule were unsuccessful. Our probe did not produce observable signals at 14.1 T, although it successfully detected a strong signal from ammonium chloride, which is highly symmetric and has a negligible quadrupolar coupling constant. Decreasing the symmetry of the nuclear-shielding environment broadens the resonance signal. The Larmor frequency of ^14^N at this field strength is only 42 MHz. In many cases, it is difficult to detect the ^14^N signal from nitrogen atoms in amide groups, which typically exhibit quadrupolar couplings of around 3.2 MHz. Furthermore, tuning for low-γ nuclei like ^14^N can be significantly affected by the quality of chip capacitors used in the probe. Unfortunately, despite our efforts, no nitrogen signal was observed from the target compound crystals under these conditions, as the resonance peaks are broadened into the baseline beyond the limit of detection.

## Theory

The NMR rotating frame Hamiltonian of an isolated quadrupolar nucleus, considered in the Zeeman interaction frame and excluding any dipolar or J-coupling interactions, includes contributions from chemical shielding and quadrupolar interactions and is expressed as^25^

1 $${Ĥ}_{\mathrm{Rot}}^{\mathrm{Total}}={Ĥ}_{Q}^{\left(1\right)}+{Ĥ}_{Q}^{\left(2\right)}+{Ĥ}_{\mathrm{CSA}}^{\left(1\right)}$$

with

2 $${Ĥ}_{Q}^{\left(1\right)}=\chi_{Q}R_{2,0}^{Q}\left\{3I_{z}^{2}-I\left(I+1\right)\right\}\left(∵\chi_{Q}=\frac{eQ}{2I\left(2I-1\right)\hbar }\right)$$

3 $$\begin{array}{ll} {Ĥ}_{Q}^{\left(2\right)} & =\frac{1}{2\omega_{0}}\chi_{Q}^{2}[R_{2,-1}^{Q}R_{2,1}^{Q}I_{z}\left\{4I\left(I+1\right)-8I_{z}^{2}-1\right\}\\ & +R_{2,-2}^{Q}R_{2,2}^{Q}I_{z}\{2I\left(I+1\right)-2I_{z}^{2}-1\}] \end{array}$$

and

$${Ĥ}_{CSA}^{\left(1\right)}=\left(\delta_{\mathrm{iso}}+R_{2,0}^{CSA}\right)\gamma B_{o}I_{z}$$ 4

In the above equations, ${Ĥ}_{Q}^{\left(1\right)}$ , ${Ĥ}_{Q}^{\left(2\right)}$ , and ${Ĥ}_{CSA}^{\left(1\right)}$ represent the first-order quadrupolar interaction, second-order quadrupolar interaction, and first-order chemical shift interaction, respectively. The term δ_iso_ denotes the isotropic chemical shift, and the components $R_{2,\lambda }^{\xi }$ (with ξ = CSA or Q, and λ = 2, 1, 0, −1, or −2) represent the spatial part of tensors defined in the laboratory (rotating) frame. Here, *I* is the nuclear spin quantum number, *I*_*z*_ is the z-component of the angular momentum operator, *eQ* is the nuclear quadrupole moment, γ is the gyromagnetic ratio, and ω_0_ is the nuclear Larmor frequency. The tensor components $R_{2,\lambda }^{\xi }$ , defined in the laboratory frame, can be related to the corresponding $G_{2,\lambda }^{\xi }$ tensor components defined in the goniometer-tenon frame through a single-step tensor rotation using the polar angle θ and an azimuthal angle ϕ according to^19,26^

$$\mathrm{Goniometer frame}\overset{\left(\phi ,\theta ,0^{◦}\right)}{\to }\mathrm{Laboratory frame}\;\left(B_{o}\;\mathrm{along the}\;\mathrm{z‐}\mathrm{axis}\right)$$ 5

After explicitly performing this transformation and reexpressing $G_{2,\lambda }^{\xi }$ defined in the polar coordinate frame into $G_{\mathrm{mn}}^{\xi }$ (where m and n are *x*, *y*, or *z*) in the Cartesian frame for easier interpretation, the expression for $R_{2,\lambda }^{\xi }\mathrm{and}R_{2,\lambda }^{\xi }R_{2,\lambda \prime }^{\xi }$ can be written as follows:

6 $$\delta_{\mathrm{iso}}=\frac{1}{3}\left(G_{zz}^{CSA}+G_{xx}^{CSA}+G_{yy}^{CSA}\right)$$

$$\begin{array}{cc} R_{2,0}^{CSA} & =\frac{1}{2}\left[\frac{1}{3}\left(2G_{zz}^{CSA}-G_{xx}^{CSA}-G_{yy}^{CSA}\right)(3\cos^{2}\theta -1)+\left(G_{xz}^{CSA}+G_{zx}^{CSA}\right)\sin2\theta \cos\phi +(G_{yz}^{CSA}+G_{zy}^{CSA})\sin2\theta \sin\phi +\left(G_{xx}^{CSA}-G_{yy}^{CSA}\right)\sin^{2}\theta \cos2\phi +(G_{xy}^{CSA}+G_{yx}^{CSA})\sin^{2}\theta \sin2\phi \right] \end{array}$$ 7

$$\begin{array}{cc} R_{2,0}^{Q} & =\frac{1}{4}[G_{zz}^{Q}\left(3\cos^{2}\theta -1\right)+\left(G_{xz}^{Q}+G_{zx}^{Q}\right)\sin2\theta \cos\phi +\left(G_{yz}^{Q}+G_{zy}^{Q}\right)\sin2\theta \sin\phi +\left(G_{xx}^{Q}-G_{yy}^{Q}\right)\sin^{2}\theta \cos2\phi +\left(G_{xy}^{Q}+G_{yx}^{Q}\right)\sin^{2}\theta \sin2\phi ] \end{array}$$ 8

$$\begin{array}{ll} R_{2,-1}^{Q}R_{2,1}^{Q} & =-[\frac{3}{4}G_{zz}^{Q}\sin2\theta -G_{xz}^{Q}\cos2\theta \cos\phi -G_{yz}^{Q}\cos2\theta \sin\phi \\ & -{\frac{1}{4}(G_{xx}^{Q}-G_{yy}^{Q})\sin2\theta \cos2\phi -\frac{1}{2}G_{xy}^{Q}\sin2\theta \sin2\phi ]}^{2}\\ & -[G_{xz}^{Q}\cos\theta \sin\phi -G_{yz}^{Q}\cos\theta \cos\phi +{\frac{1}{2}(G_{xx}^{Q}-G_{yy}^{Q})\sin\theta \sin2\phi -G_{xy}^{Q}\sin\theta \cos2\phi ]}^{2} \end{array}$$ 9

$$\begin{array}{ll} R_{2,-2}^{Q}R_{2,2}^{Q} & =[\frac{3}{4}G_{zz}^{Q}\sin^{2}\theta -\frac{1}{2}G_{xz}^{Q}\sin2\theta \cos\phi -\frac{1}{2}G_{yz}^{Q}\sin2\theta \sin\phi \\ & +{\frac{1}{4}(G_{xx}^{Q}-G_{yy}^{Q})(1+\cos^{2}\theta )\cos2\phi +\frac{1}{2}G_{xy}^{Q}\left(1+\cos^{2}\theta \right)\sin2\phi ]}^{2}\\ & +[G_{xz}^{Q}\sin\theta \sin\phi -G_{yz}^{Q}\sin\theta \cos\phi -{\frac{1}{2}(G_{xx}^{Q}-G_{yy}^{Q})\cos\theta \sin2\phi +G_{xy}^{Q}\cos\theta \cos2\phi ]}^{2} \end{array}$$ 10

The relevant expressions used to interpret the -*x* ^*T*^ rotation (θ = -Θ; ϕ = π/2), *y* ^*T*^ rotation (θ = Θ; ϕ = 0), and -*z* ^*T*^ rotation (θ = π/2; ϕ= -Θ) patterns for the transitions of ^11^B (*I* = 3/2) can be derived from the equations above. Here, Θ represents the rotational angle applied experimentally by rotating the crystal about an axis that is oriented 90^◦^ relative to the external magnetic field (see Figure 2). For the central transition, all three rotation patterns can be simplified to the following expressions, incorporating the first-order CSA and second-order quadrupolar contributions:

$$\nu_{|\frac{1}{2}\rangle ↔|-\frac{1}{2}\rangle }^{CSA}=A^{CSA}+B^{CSA}\cos2\Theta +C^{CSA}\sin2\Theta$$ 11

$$\nu_{|\frac{1}{2}\rangle ↔|-\frac{1}{2}\rangle }^{Q2}=A^{Q}+B^{Q}\cos2\Theta +C^{Q}\sin2\Theta +D^{Q}\cos4\Theta +E^{Q}\sin4\Theta$$ 12

where α ∈ {-*x* ^*T*^, *y* ^*T*^, -*z* ^*T*^} and the coefficients $\Gamma_{m}^{\xi }$ (with Γ = *A*, *B*, *C*,···,*E*) are defined in terms of the $G_{\mathrm{mn}}^{\xi }$ tensor components, as given in eqs 6–10. Note that the dominant first-order quadrupolar Hamiltonian vanishes for symmetric transitions such as $\left|\frac{1}{2}\right\rangle ↔\left|-\frac{1}{2}\right\rangle$ , due to its quadratic dependence on $I_{z}^{2}$ .

The CSA and quadrupolar tensor parameters $G_{\mathrm{mn}}^{\xi }$ (3 × 3 matrices) in the goniometer-tenon frame are obtained by performing least-squares curve fitting of the experimentally acquired -*x* ^*T*^, *y* ^*T*^, and -*z* ^*T*^ rotation patterns using the expressions above. Subsequently, the tensor parameters in the principal axis frames (PAFs) of both the CSA and the quadrupolar tensors can be obtained by matrix diagonalization. These principal components represent unique molecular properties and provide a generalized framework for describing the corresponding NMR tensor interactions. In the principal axis frame (PAF), the CSA and quadrupolar tensors can be represented as follows:

13 $$\delta_{\mathrm{iso}}=\frac{1}{3}\left(\delta_{11}+\delta_{22}+\delta_{33}\right)$$

14 $$\delta_{\mathrm{CS}}=\delta_{33}-\delta_{\mathrm{iso}}$$

15 $$\eta_{\mathrm{CS}}=\frac{\delta_{22}-\delta_{11}}{\delta_{\mathrm{CS}}}$$

with the convention^25^

16 $$\left|\delta_{33}-\delta_{\mathrm{iso}}\right|\geq \left|\delta_{11}-\delta_{\mathrm{iso}}\right|\geq \left|\delta_{22}-\delta_{\mathrm{iso}}\right|$$

for CSA and

17 $$C_{Q}=\frac{eQ\cdot V_{33}}{h}$$

18 $$\eta_{Q}=\frac{V_{22}-V_{11}}{V_{33}}$$

with the convention

19 $$\left|V_{33}\right|\geq \left|V_{11}\right|\geq \left|V_{22}\right|$$

for the quadrupolar tensors. Here, $\delta_{\mathrm{mn}}^{CS}$ and $V_{\mathrm{mn}}^{Q}$ denote the CSA tensor and electric field gradient (EFG) tensor, respectively, each defined in its corresponding PAF, where the subscript 11 corresponds to *xx*, 22 to *yy*, and 33 to *zz* in eqs 6–10.

Tensors defined in the principal axis frame (PAF) and the goniometer-tenon frame are related through unitary transformation. Typically, the PAF of the quadrupolar interaction is used as a common reference frame, through which the PAF of the CSA tensor—and, if available, the crystal axis frame determined by X-ray crystallography—can also be expressed. By using the crystal axis frame as the shared reference, both the quadrupolar and CSA PAF can be transformed to the goniometer-tenon frame, where the crystal is physically mounted using the following tensor transformations:

20 $$\mathrm{PAF}\left(\mathrm{CSA}\right)\overset{\left(a,b,c\right)}{\to }\mathrm{PAF}\left(Q\right)\overset{\left(\zeta ,\lambda ,\nu \right)}{\to }\mathrm{Crystal axis frame}\overset{\left(\alpha ,\beta ,\gamma \right)}{\to }\underset{\left(\mathrm{Rotating frame}\right)}{\mathrm{Goniometer frame}}$$

Two tensors A and B that are related by three consecutive passive rotations involving a Euler’s angle set (α_1_, β_1_, γ_1_) can be expressed by a unitary transformation as

21 $$B=R\left(\alpha_{1},\beta_{1},\gamma_{1}\right)AR^{-1}\left(\alpha_{1},\beta_{1},\gamma_{1}\right)$$

where *R*(α_1_, β_1_, γ_1_), the rotation matrix, is defined as^26–28^

22 $$R\left(\alpha_{1},\beta_{1},\gamma_{1}\right)=\left(\begin{array}{ccc} c\alpha_{1}c\beta_{1}c\gamma_{1}-s\alpha_{1}s\gamma_{1} & s\alpha_{1}c\beta_{1}c\gamma_{1}+c\alpha_{1}s\gamma_{1} & -s\beta_{1}c\gamma_{1}\\ -c\alpha_{1}c\beta_{1}s\gamma_{1}-s\alpha_{1}c\gamma_{1} & -s\alpha_{1}c\beta_{1}s\gamma_{1}+c\alpha_{1}c\gamma_{1} & s\beta_{1}s\gamma_{1}\\ c\alpha_{1}s\beta_{1} & s\alpha_{1}s\beta_{1} & c\beta_{1} \end{array}\right)$$

where *c*θ ≡ cosθ and *s*θ ≡ sinθ.

## Experimental Details

### Synthesis of (+,+)- and (-,-)-8-HQ­(ipc)_2_B

The fluorescent yellow-green (+,+) and (-,-) enantiomers of 8-HQ­(ipc)_2_B were individually synthesized following the procedure previously described^29^ from the corresponding *B*-methoxydiisopinocampheyl-borane enantiomer (Santa Cruz Biotechnology) and 8-hydroxy-quinoline (99% purity, Thermo Scientific). The reaction and conditions are given in Scheme 1. The crude product was collected by vacuum filtration. The molar optical rotations of the (+,+) and (-,-) enantiomers were measured by polarimetry in acetone solution at 589 nm to be 58 ± 5^◦^ *mol* ^–1^ *cm* ^–1^ and −53 ± 5^◦^ *mol* ^–1^ *cm* ^–1^, respectively, Figure 1.

**Scheme 1:**
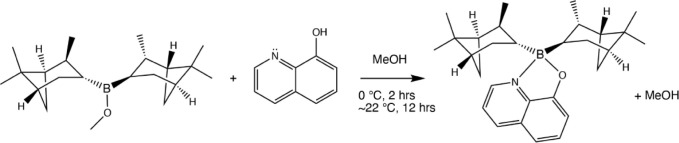
Synthesis of 8-HQ­(ipc)_2_B

**Figure 1:**
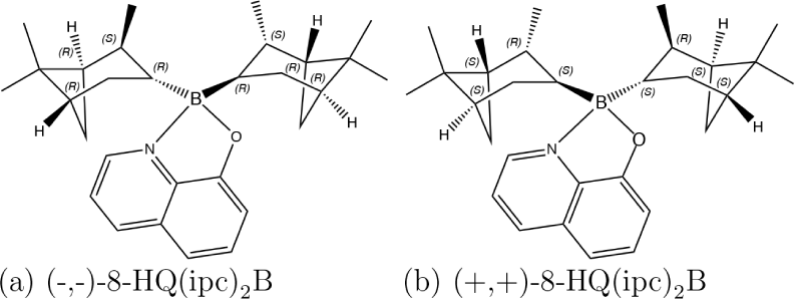
Structures of the 8-HQ­(ipc)_2_B enantiomers with absolute configurations for each chiral center used in this study.

Single crystals for each enantiomer were prepared separately by dissolving the product in methanol, heating to 40^◦^C, filtering, cooling, and placing in a 4^◦^C refrigerator to grow crystals using the slow evaporation method. After about 4–5 weeks, the crystals were harvested. The crystals were quickly washed with cold methanol to remove any surface impurities and stored at room temperature away from light as photosensitivity of the compound was observed. Single crystals for the study were screened using the cross-polarized light microscopy technique^30^ to check for twinned and polycrystals; samples were observed while rotating them under crossed polarizers to verify uniform light transmission. The single crystals selected for the study had dimensions of approximately 3 × 3 × 2 mm^3^ for (-,-)-8-HQ­(ipc)_2_B and 3 × 2 × 1 mm^3^ for (+,+)-8-HQ­(ipc)_2_B. The single crystals were glued to a tenon plate using epoxy resin.

### Single-Crystal X-ray Diffraction

The lattice structure parameters were determined using powder X-ray diffraction (XRD) analysis at the Electron Microbeam Analysis Laboratory (EMAL), University of Michigan. Powdered (-,-) and (+,+)-8-HQ­(ipc)_2_B samples were analyzed in reflection mode in Bragg–Brentano geometry on a Rigaku Ultima IV X-ray diffractometer. The Cu anode X-ray beam (40 kV, 44 mA) was filtered by a 20 μm thick nickel foil to remove Cu *K*β, giving monochromatic Cu *K*α X-rays. The divergence, scattering, and receiving slits were set at 2/3^◦^, 2/3^◦^, and 0.6 mm, respectively. The scanning 2θ range was from 5^◦^ to 70^◦^ with a step size of 0.02^◦^ at a scan rate of 1^◦^ min^–1^. The unit cell parameters (*a*, *b*, *c*, α, β, γ) were recovered by assigning the appropriate triple of Miller indices (*hkl*) to each observed interplanar spacing (*d*_*hkl*_). The indexing process was performed using EXPO2014 ^31^ via the N-TREOR09 program,^32^ the evolution of the N-TREOR software.^33^

**Table 1:** Crystallographic Data for 8-HQ­(ipc)_2_B

Empirical formula	C_29_H_40_BNO
Formula weight	429.43
Temperature	293 (2) K
Crystal system	Monoclinic
Space group	5, C121
Cell lengths	*a* = 20.333 (4) Å, *b* = 10.955 (2) Å, *c* = 12.550 (3) Å
Cell angles	α = γ = 90.0 ^◦^, β = 109.836 (13)^◦^
Z	4
Cell volume	2629.5 (10) Å^3^

**Figure 2:**
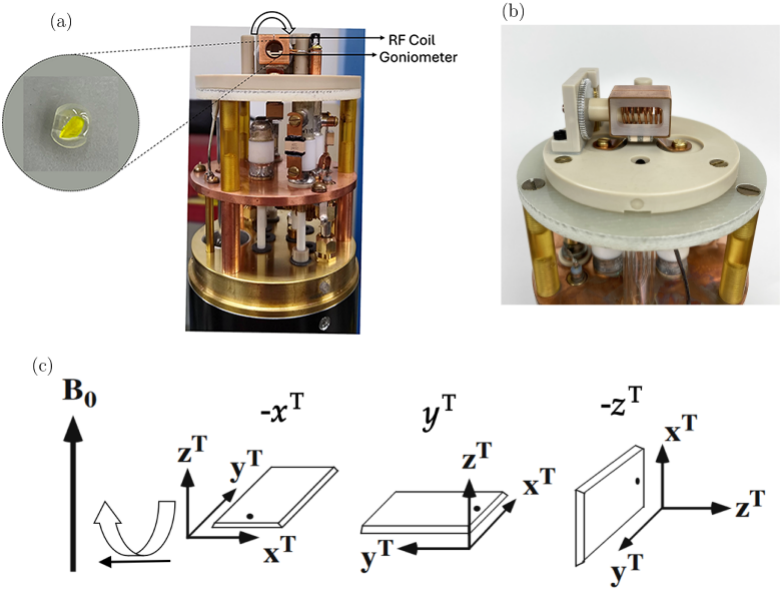
(a) Photograph of the front view of the probe. The arrow shows the rotation of the goniometer inside the RF coil. Inset shows (+,+)-8-HQ­(ipc)_2_B crystal glued to the tenon. The tenon is mounted on three different orientations in the goniometer so that three orthogonal rotations of the sample could be achieved. (b) Photograph of the side view of the probe showing the goniometer mechanism and probe coils. (c) Rotation of tenon around an axis that is perpendicular to magnetic field. The three mutually perpendicular rotations are achieved by mounting the tenon plate into dovetails for -*x* ^*T*^, *y* ^*T*^, and -*z* ^*T*^ rotations (subfigure reproduced from ref 26. Available under a CC BY 4.0 license. Copyright Vosegaard, T.).

**Table 2:** Average Root-Mean-Square Deviations in Nuclear Positions after Optimization of (-,-)-8-HQ­(ipc)_2_B from the Literature Crystal Structure

Nucleus	# centers	RMS deviation/Å
H	160	0.042
B	4	0.009
C	116	0.035
N	4	0.028
O	4	0.022
All	288	0.038

**Table 3:** Calculated Quadrupolar (in MHz) and Chemical Shielding (CS) Tensors (in ppm), Rounded to Three Decimal Places, for One of the Magnetically Equivalent ^11^B Pairs in Enantiomers of 8-HQ­(ipc)_2_B

	Enantiomer	
Tensor	(-,-)-8-HQ(ipc)_2_B	(+,+)-8-HQ(ipc)_2_B
Quadrupolar	$$\left(\begin{array}{ccc} -0.273 & 1.787 & 1.062\\ 1.787 & -0.056 & 0.253\\ 1.062 & 0.253 & 0.329 \end{array}\right)$$	$$\left(\begin{array}{ccc} -0.273 & -1.784 & 1.063\\ -1.784 & -0.059 & -0.254\\ 1.063 & -0.254 & 0.331 \end{array}\right)$$
Total CS	$$\left(\begin{array}{ccc} 90.564 & 4.067 & 1.696\\ -2.082 & 84.482 & 6.711\\ -4.059 & 2.216 & 76.986 \end{array}\right)$$	$$\left(\begin{array}{ccc} 90.594 & -4.048 & 1.716\\ 2.106 & 84.496 & -6.704\\ -4.052 & -2.216 & 77.027 \end{array}\right)$$
Isotropic CS	$$\left(\begin{array}{ccc} 84.011 & 0 & 0\\ 0 & 84.011 & 0\\ 0 & 0 & 84.011 \end{array}\right)$$	$$\left(\begin{array}{ccc} 84.039 & 0 & 0\\ 0 & 84.039 & 0\\ 0 & 0 & 84.039 \end{array}\right)$$
Symmetric CS	$$\left(\begin{array}{ccc} 90.564 & 0.993 & -1.182\\ 0.993 & 84.482 & 4.464\\ -1.182 & 4.464 & 76.986 \end{array}\right)$$	$$\left(\begin{array}{ccc} 90.594 & -0.971 & -1.168\\ -0.971 & 84.496 & -4.460\\ -1.168 & -4.460 & 77.027 \end{array}\right)$$
Antisymmetric CS	$$\left(\begin{array}{ccc} 0 & 3.075 & 2.877\\ -3.075 & 0 & 2.247\\ -2.877 & -2.247 & 0 \end{array}\right)$$	$$\left(\begin{array}{ccc} 0 & -3.077 & 2.884\\ 3.077 & 0 & -2.244\\ -2.884 & 2.244 & 0 \end{array}\right)$$

**Table 4:** Calculated Quadrupolar (in MHz) and Chemical Shielding (CS) Tensors (in ppm), Rounded to Three Decimal Places, for One of the Magnetically Equivalent ^14^N Pairs in Enantiomers of 8-HQ­(ipc)_2_B

	Enantiomer	
Tensor	(-,-)-8-HQ(ipc)_2_B	(+,+)-8-HQ(ipc)_2_B
Quadrupolar	$$\left(\begin{array}{ccc} 0.670 & -0.054 & -0.729\\ -0.054 & 0.903 & 0.008\\ -0.729 & 0.008 & -1.573 \end{array}\right)$$	$$\left(\begin{array}{ccc} 0.671 & 0.055 & -0.730\\ 0.055 & 0.903 & -0.009\\ -0.73 & -0.009 & -1.575 \end{array}\right)$$
Total CS	$$\left(\begin{array}{ccc} 54.663 & -171.000 & -41.720\\ -176.101 & -10.025 & 65.249\\ -50.172 & 59.397 & -101.142 \end{array}\right)$$	$$\left(\begin{array}{ccc} 54.548 & 171.093 & -41.663\\ 176.228 & -10.211 & -65.303\\ -50.105 & -59.473 & -101.176 \end{array}\right)$$
Isotropic CS	$$\left(\begin{array}{ccc} -18.835 & 0 & 0\\ 0 & -18.835 & 0\\ 0 & 0 & -18.835 \end{array}\right)$$	$$\left(\begin{array}{ccc} -18.946 & 0 & 0\\ 0 & -18.946 & 0\\ 0 & 0 & -18.946 \end{array}\right)$$
Symmetric CS	$$\left(\begin{array}{ccc} 54.663 & -173.551 & -45.946\\ -173.551 & -10.025 & 62.323\\ -45.946 & 62.323 & -101.142 \end{array}\right)$$	$$\left(\begin{array}{ccc} 54.548 & 173.661 & -45.884\\ 173.661 & -10.211 & -62.388\\ -45.884 & -62.388 & -101.176 \end{array}\right)$$
Antisymmetric CS	$$\left(\begin{array}{ccc} 0 & 2.551 & 4.226\\ -2.551 & 0 & 2.926\\ -4.226 & -2.926 & 0 \end{array}\right)$$	$$\left(\begin{array}{ccc} 0 & -2.567 & 4.221\\ 2.567 & 0 & -2.915\\ -4.221 & 2.915 & 0 \end{array}\right)$$

**Figure 3:**
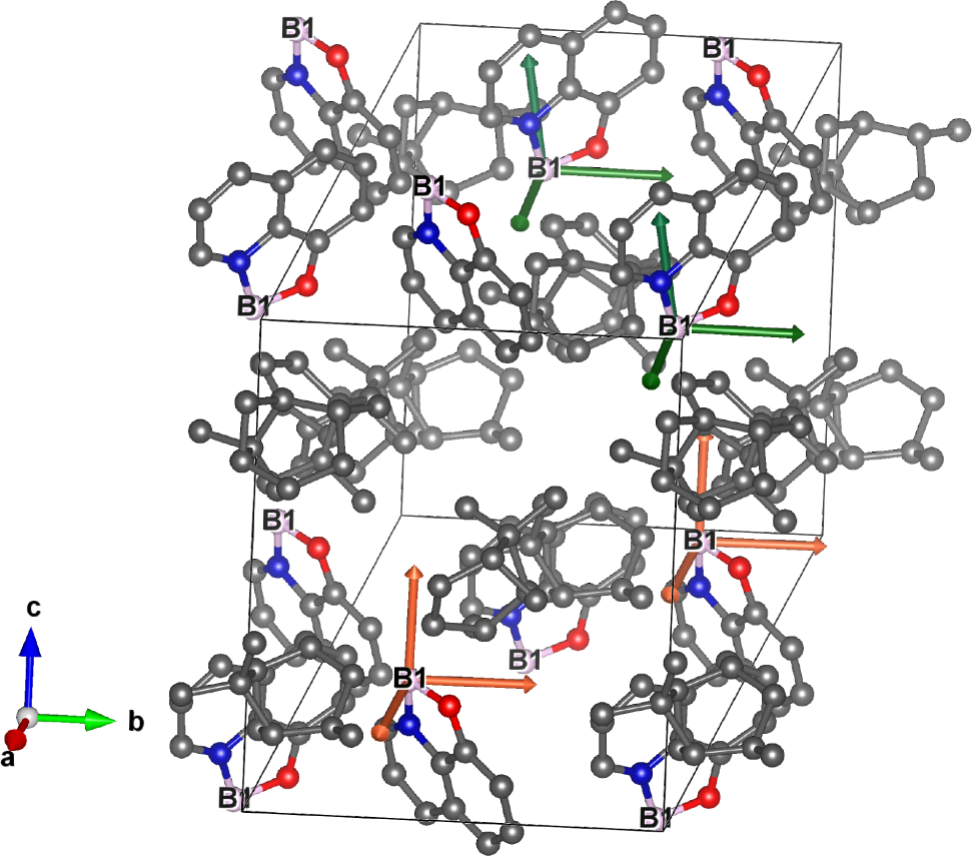
Monoclinic crystal structure of 8-HQ­(ipc)_2_B with crystallographically equivalent B nuclei labeled as B1. The sets of magnetically nonequivalent B nuclei and their chemical shielding tensors are represented in orange and green colors. (*The selection of magnetically nonequivalent pair is arbitrary.*) The arrows represent directions and scaled magnitudes of computed chemical shielding tensors in the crystal axis system. B, C, N, and O nuclei are shown in pink, gray, blue, and red, respectively. (H nuclei omitted for clarity.).

**Figure 4:**
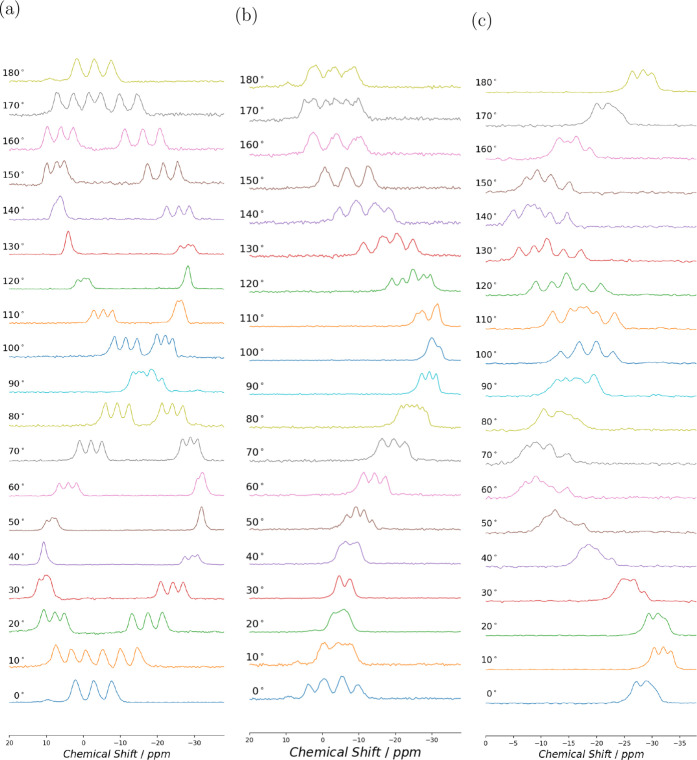
^11^B NMR spectra at 14.1 T for (-,-)-8-HQ­(ipc)_2_B. The spectra were recorded at 10^◦^ increments for rotation about (a) mounting -*x* ^*T*^, (b) mounting *y* ^*T*^, and (c) mounting -*z* ^*T*^.

**Figure 5:**
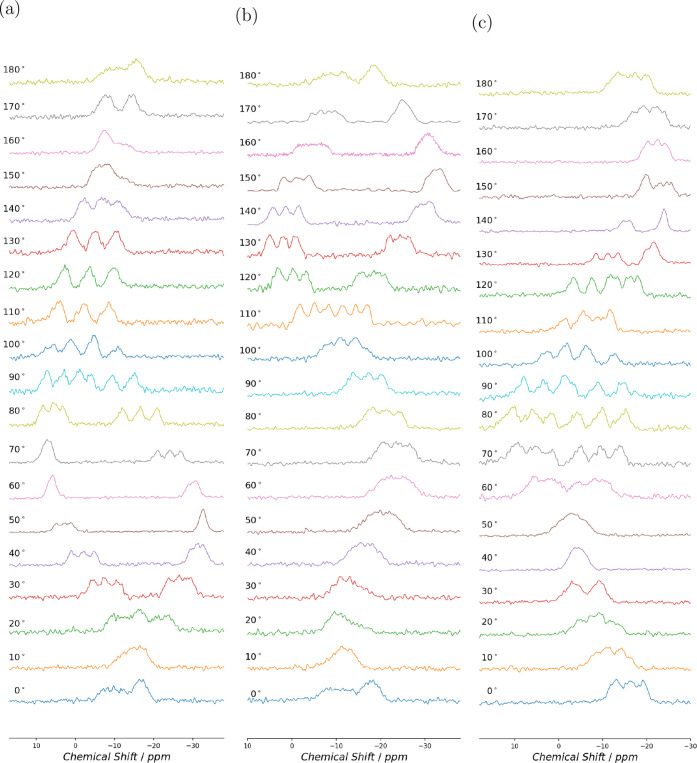
^11^B NMR spectra at 14.1 T for (+,+)-8-HQ­(ipc)_2_B. The spectra were recorded at 10^◦^ increments for rotation about (a) mounting -*x* ^*T*^, (b) mounting *y* ^*T*^, and (c) mounting -*z* ^*T.*^

**Figure 6:**
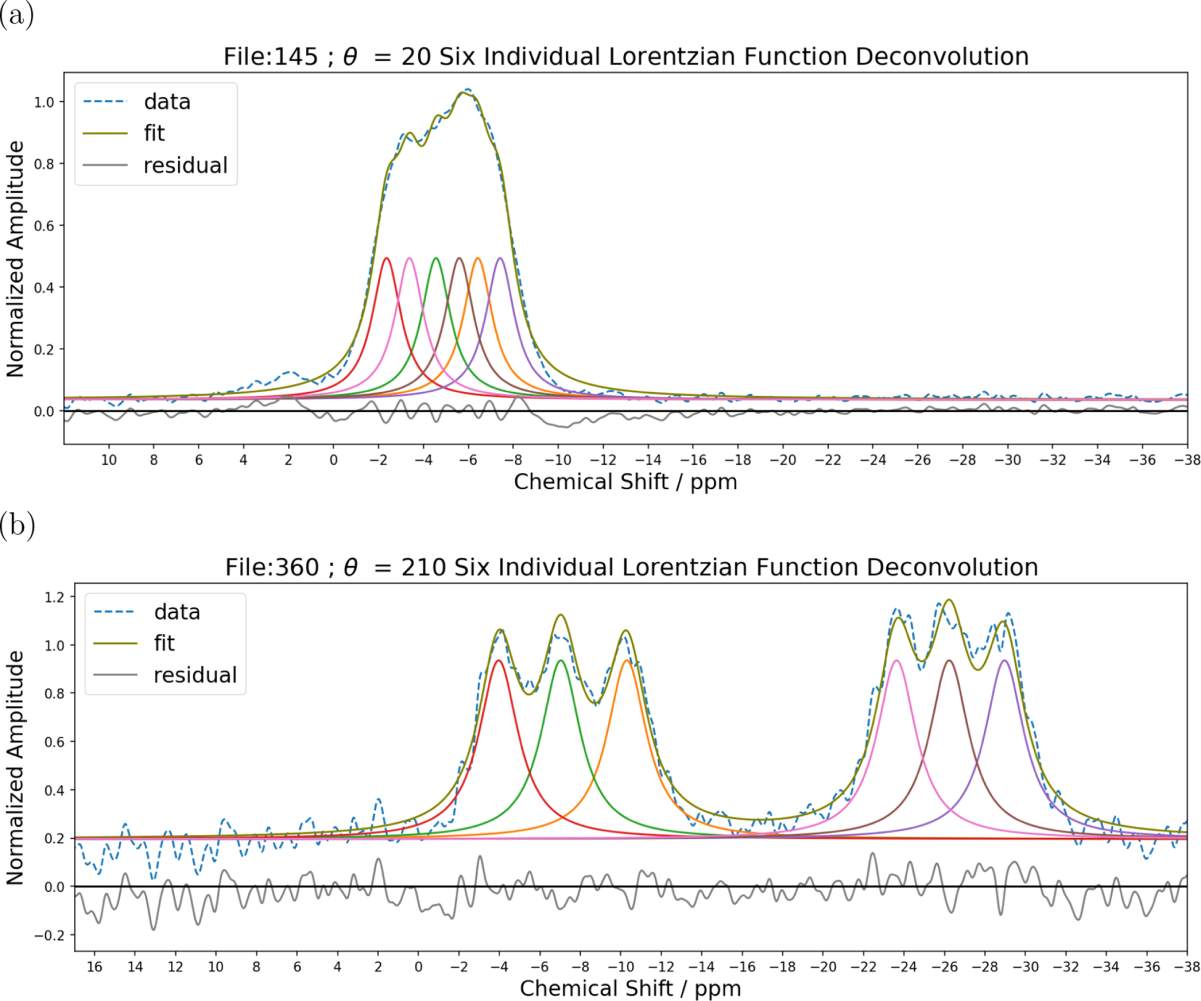
Deconvolution of two representative experimental spectra showing data and spectral reconstruction after fitting. The residuals are also shown to demonstrate the goodness of fit. (a) (-,-)-8-HQ­(ipc)_2_B rotation about *y* ^*T*^ at a 20^◦^ rotation angle and (b) (+,+)-8-HQ­(ipc)_2_B rotation about -*x* ^*T*^ at a 210^◦^ rotation angle.

**Figure 7:**
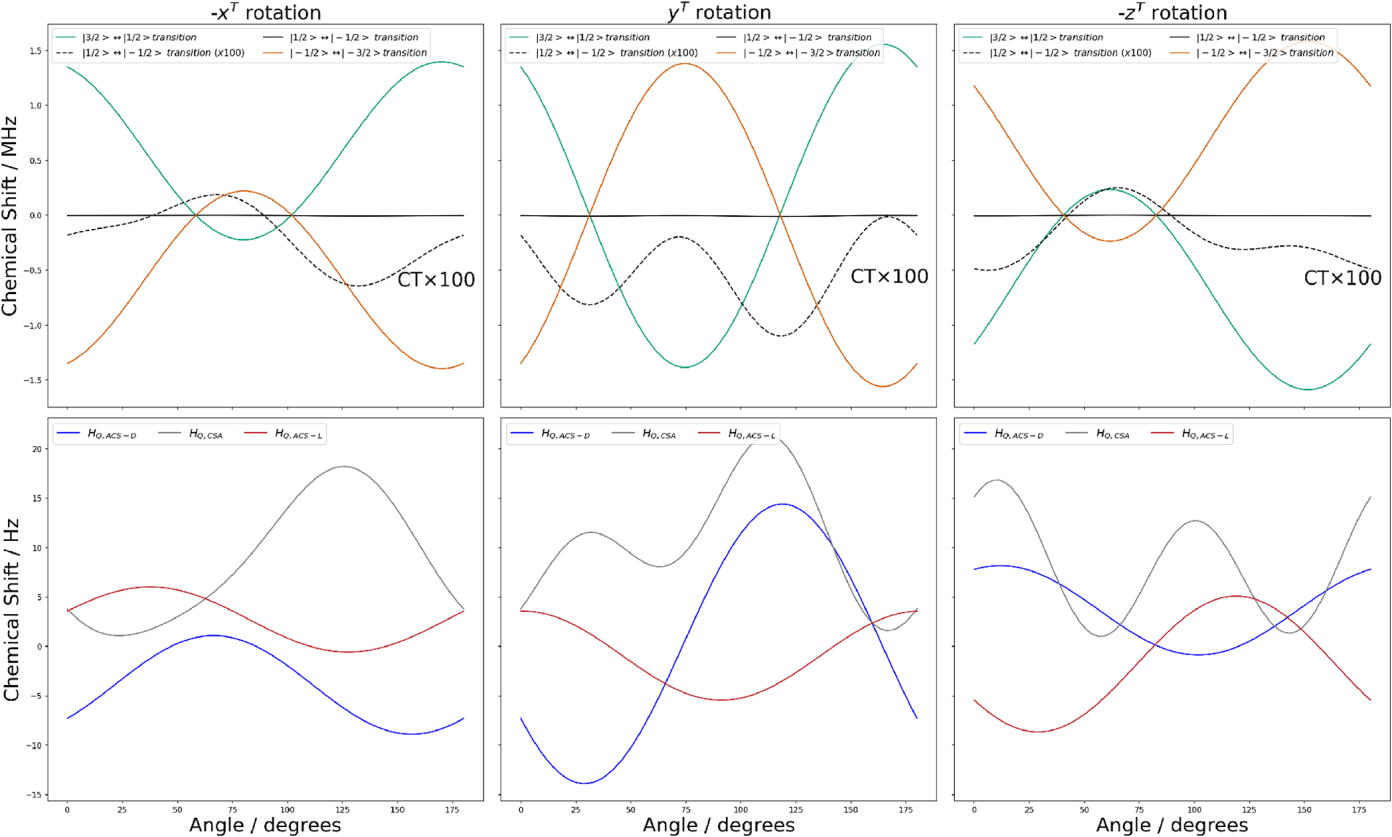
(**Top**) Simulation of ^11^B single-crystal NMR spectra for one of the magnetic site in (+,+)-8-HQ­(ipc)_2_B for rotation about the -*x* ^*T*^, *y* ^*T*^, and -*z* ^*T*^ axes. The simulation employs NMR parameters from DFT calculations and Euler angles derived from XRD analysis to relate the crystal frame to the tenon frame. Satellite transitions are shown in green $\left(\left|\frac{3}{2}\right\rangle ↔\left|\frac{1}{2}\right\rangle \right)$ , orange $\left(\left|-\frac{1}{2}\right\rangle ↔\left|-\frac{3}{2}\right\rangle \right)$ ), and the central transition in black $\left(\left|\frac{1}{2}\right\rangle ↔\left|-\frac{1}{2}\right\rangle \right)$ . The dashed black curve (CT × 100) represents the central transition magnified by a factor of 100. (Bottom) The quadrupolar–ACS for the two enantiomers (*D:* blue, *L:* red) and quadrupolar–CSA (gray) contributions calculated from addition of the simulated satellite frequencies, following ref^53^.

**Table 5:** Optimized Coefficients (in kHz) for ^11^B Rotation Data of (-,-)-8-HQ­(ipc)_2_B (up to 3 Significant Figures)

Mounting	Nucleus	*A*	*B*	*C*	*D*	*E*
-*x* ^*T*^	1	–2.24	1.43	–3.52	0.220	0.00429
*y* ^*T*^		–2.63	2.46	0.272	–0.402	–0.526
-*z* ^*T*^		–3.47	–1.14	–0.574	–0.940	–1.09
-*x* ^*T*^	2	–1.74	1.30	3.55	–0.0424	–0.0274
*y* ^*T*^		–2.75	2.59	1.01	–0.326	–0.558
-*z* ^*T*^		–3.26	–1.19	–0.629	–0.957	–1.13

**Table 6:** Optimized Coefficients (in kHz) for ^11^B Rotation Data of (+,+)-8-HQ­(ipc)_2_B (up to 3 Significant Figures)

Mounting	Nucleus	*A*	*B*	*C*	*D*	*E*
-*x* ^*T*^	1	–2.77	–0.0218	–2.61	0.911	0.405
*y* ^*T*^		–3.85	–0.0658	0.820	0.510	1.54
-*z* ^*T*^		–1.87	–1.93	2.05	0.605	–0.0456
-*x* ^*T*^	2	–0.903	–1.81	0.662	–0.374	–0.494
*y* ^*T*^		–2.05	0.689	–1.91	–0.481	0.490
-*z* ^*T*^		–2.04	–0.698	0.778	–0.289	0.806

**Table 7:** Experimental and Calculated Quadrupolar Couplings, Chemical Shift Anisotropies, Isotropic Chemical Shift, and Relative Orientations of the Two Tensors for ^11^B and ^14^N Nuclei in (+,+)-8-HQ­(ipc)_2_B and (-,-)-8-HQ­(ipc)_2_B

	Compound	Nucleus	|*C*_*Q*_|/MHz	η_*Q*_	δ_CS_/ppm	η_CS_	δ_iso_ ^a^/ppm	*a*/deg	*b*/deg	*c*/deg
**Experimental**	(+,+)-8-HQ(ipc)_2_B	^11^B	2.25 ± 0.07	0.90 ± 0.07	-9.3 ± 0.8	0.27 ± 0.18	–8^b^	81 ± 22	91 ± 4	71 ± 4
	(-,-)-8-HQ(ipc)_2_B	^11^B	2.26 ± 0.12	0.78 ± 0.13	-8.5 ± 1.3	0.4 ± 0.4	–8^b^	84 ± 44	89 ± 7	92 ± 6
**Calculated PBEsol + GIPAW**	(+,+) and (-,-)-8-HQ(ipc)_2_B	^11^B	2.12	1.00	–9.2	0.46	84.01^c^	47	95	76
		^14^N	1.79	0.06	235.60	0.22	–18.83^c^	78	90	91

a Isotropic chemical shifts are relative to BF_3_OEt_2_ in CDCl_3_ at 26.9 ppm.

b Error limit could not be estimated.

c δ_iso_ = σ_reference_ - σ_sample_; for calculated values, σ_reference_ = 0.

The orientation of the single crystals was determined by using the so-called “Omega-Scan” method. Briefly, the crystal and its tenon-plate support were mounted on a thin-film stage of a Rigaku Ultima IV diffractometer with a straight edge of the plate parallel to the goniometer rotation axis. The specimen was rotated by 360^◦^ around a defined axis, for example, the surface normal (ϕ scan). The angular positions of the reflections were used to evaluate the orientation of the crystal lattice (completely described by three angles) in relation to the tenon-plate axis.

Powder XRD confirmed that the unit cells match the reported crystal structure^22^ of 8-HQ­(ipc)_2_B, given in Table 1. The surface normal and edge plane directions of the mounted crystals were used to find the orientation matrix and eventually the orientation of the crystal axis systems relative to the tenon axis systems. The Euler angles relating the crystal axis frame to the goniometer frame for the (+,+)-8-HQ­(ipc)_2_B crystal are 153.5^◦^, 153.4^◦^, and 179.9^◦^, and for the (-,-)-8-HQ­(ipc)_2_B crystal are 280.0^◦^, 45.0^◦^, and 180.0^◦^ (see the Supporting Information for details).

### Single-Crystal NMR Spectroscopy

NMR spectra for both samples were acquired using a Bruker Avance III console running Topspin 3.6 (Bruker Biospin GmbH) at the National High Magnetic Field Laboratory (NHMFL) in Tallahassee, FL. A custom-built low-E 600 MHz static HX probe (Figure 2a,b), developed at the NHMFL, was used for the measurements. The probe features a cross-coil arrangement of a 6.5 mm ID round 9-turn X-channel detection solenoid coil mounted inside and orthogonal to a low-inductance ^1^H-channel loop-gap resonator that was employed for the measurements. Initial tuning configurations were made for ^1^H–^14^N, ^1^H–^17^O, and ^1^H–^11^B. Detailed descriptions of the low-E coil and probe circuitry are available elsewhere.^34^

This probe was optimized for ^11^B detection with ^1^H decoupling and operated inside a 600 MHz (14.1 T), 89 mm bore magnet. The tenon plate, with the crystal glued on it, was mounted in the dovetail track of the goniometer and positioned within the 6 mm inner diameter loop-gap resonator-type NMR sample coil, enabling stepwise rotation to acquire single-crystal NMR spectra across defined rotation patterns. The mounting configuration was designed to allow positive rotations of the tenon about the -*x* ^*T*^, *y* ^*T*^, and -*z* ^*T*^ axes, as defined by the dovetail pattern inscribed in the goniometer.^35^

Sample rotation was driven by a worm gear arrangement, consisting of a modified 72-tooth aluminum spur gear with a 0.600 in. pitch diameter and 0.062 in. face width (manufacturer part number LAA-K1–72 from Nordex, Inc.). A brass worm screw was machined to rotate the spur gear, with a major diameter of 0.125 in. and 40 threads per inch pitch. Each full rotation of the worm screw turns the goniometer stage by 5^◦^. A counterdial mounted to the base of the probe allows the user to read angular adjustments in 0.1^◦^ increments.

Three goniometer stages, providing *x*, *y*, and *z* sample orientation, were machined from unfilled PEEK polymer and attached with screws to the spur gear so that the sample rotates close to the detection solenoid axis and at the middle turn of the coil. Each goniometer stage features a mortise slot into which the sample tenon mount can firmly inserted. The sample tenon was machined from Rexolite 1422 cross-linked polystyrene, which exhibits excellent dielectric properties and RF transparency at microwave frequencies. Rexolite also features good bonding properties, so that the sample can be readily epoxied to the tenon mount, which can accommodate a maximum sample size of 4.4 × 4.4 × 2.5 mm^3^. Figure 2 shows the probe, the single-crystal sample mounted on the tenon, and the three orthogonal tenon rotations relative to the magnetic field, which vary depending on how the plate is mounted in the goniometer.

The rotations were performed from 0^◦^ to 360^◦^ in 10^◦^ increments using a worm gear mechanism, controlled by adjusting a micrometer scale located at the bottom of the probe and connected to the gear via a shaft. To minimize backlash error, all rotations were carried out in a single direction. The 90° pulse lengths were 2.5 μ*s* for the ^11^B channel and 3.2 μ*s* for the ^1^H channel, respectively. Both the low-frequency (^11^B) and high-frequency (^1^H) channels were manually retuned and rematched at least every other angular increment. Any retuning was minor, and was required, we believe, due to small changes in the filling factor of the sample and mount in the probe detection volume. All spectra were acquired at room temperature (≈22^◦^ C) with air cooling. For both enantiomers (+,+) and (-,-)-8-HQ­(ipc)_2_B, spectra were recorded along each rotation axis using a spectral width of 40 kHz and an acquisition time of 20 *ms*. Between 128 and 1024 transients were collected per spectrum with an acquisition delay time of 1 *s*, as needed to maintain a consistent signal-to-noise ratio (SNR). The rotation patterns were verified by observing smooth curves connecting the recorded resonance frequencies and by comparing the spectra at the starting and ending rotational positions as well as key check points such as 0^◦^(-*x* ^*T*^) = 0^◦^(*y* ^*T*^), 90^◦^(-*x* ^*T*^) = 90^◦^(-*z* ^*T*^), and 0^◦^ (-*z* ^*T*^) = 90^◦^(*y* ^*T*^). Spectral calibration was carried out using an external reference sample of boron trifluoride etherate (BF_3_OEt_2_) in CDCl_3_ with an ^11^B chemical shift of 26.9 ppm.

### Computational Modeling

The literature X-ray crystal structure^22^ of (-,-)-8-HQ­(ipc)_2_B was used as the basis for the analogous computed structure. As the atom positions in the X-ray structure are somewhat uncertain—heavy atoms’ positions occupy a thermal ellipsoid and the hydrogens’ positions are inferred—the starting X-ray structure was optimized under 3-dimensional periodic boundary conditions using a plane-wave density functional approach, as implemented in the CASTEP software package,^36^ using the PBEsol exchange-correlation functional^37–39^ with “precise″ basis precision and automatic finite-basis-set correction.^40^ Three k-points were used for Brillouin-zone sampling.^41^ During optimization, the unit-cell parameters were fixed at the literature values, and the nuclear positions allowed to relax.^42–44^ The minor differences between the literature and optimized structures are summarized as root-mean-square displacements, as shown in Table 2. The structure for the (+,+) enantiomer was generated from that of the (-,-) structure by reflection of the coordinates across a plane perpendicularly bisecting the *v*-axis. The electric field gradients^45^ and nuclear magnetic shielding tensors^46^ of both crystal enantiomers were calculated using ultra-soft pseudopotentials^47,48^ using the GIPAW (Gauge Including Projector Augmented Waves) approach^23,49^ as implemented in the CASTEP-NMR package. The calculated tensors are given in Tables 3 and 4.

The NMR parameters were then calculated using eqs 13–16 and eqs 17–19 and the mutual orientation of the two tensors using eq 21. Spin–Spin coupling calculations^50^ were carried out with the nitrogen center as the perturbing nucleus, specifically to probe the ^11^B–^14^N interaction. A single center was sufficient as all ^11^B–^14^N bonds are symmetrically related and chemically identical.

## Results and Discussion

As shown in Figure 3, the unit cell for 8-HQ­(ipc)_2_B has one crystallographically equivalent site with two pairs of magnetically nonequivalent boron nuclei related by screw symmetry. Thus, two resonance frequencies from magnetically nonequivalent boron sites are expected. Also shown in the figure are the calculated orientations of the chemical shielding anisotropy tensors of the boron sites.

Since each boron nucleus is bonded to a ^14^N (*I* = 1, natural abundance ∼ 99.6%), most orientations of an 8-HQ­(ipc)_2_B single crystal in an external magnetic field are expected to show a 1:1:1 spin–spin coupled triplet for the central transition $\left(\left|\frac{1}{2}\right\rangle ↔\left|-\frac{1}{2}\right\rangle \right)$ from each magnetically nonequivalent sites in the ^11^B NMR spectra. The single-crystal ^11^B NMR spectra for (-,-)-8-HQ­(ipc)_2_B rotation about the -*x* ^*T*^, *y* ^*T*^, and -*z* ^*T*^ axes are shown in Figure 4, and those for (+,+)-8-HQ­(ipc)_2_B are shown in Figure 5. While the spectra were recorded for 0^◦^–360^◦^ rotation, only data from 0^◦^–180^◦^ are shown.

The assignment for individual resonances in each spectrum required for the rotation plots (see Figures 8 and 9) was accomplished using an in-house deconvolution program written in Python employing the least-squares cost function from the iminuit minimization software package^51^ (see the Supporting Information). The program was used to fit the spectra with six Lorentzian functions after baseline adjustment, and phase correction was completed using TopSpin version 4.3.0 (Bruker Biospin GmbH). The deconvolution was facilitated by first fitting the spectrum as two groups having three Lorentzian functions. The parameters obtained were used as input to then fit the spectrum with six individual Lorentzian functions such that the area under the curve for each individual function was the same. The goodness of fit was evaluated by residuals analysis (see Figure 6).

The correlations of the rotation plots and peak assignments were assisted by the fact that the NMR chemical shift frequency remains the same for certain pairs of crystal orientations

$$\mathrm{Mounting ‐}x^{T}\left(\Theta =0^{◦}\right)\equiv \mathrm{Mounting}\;y^{T}\left(\Theta =0^{◦}\right)$$ 23

$$\mathrm{Mounting ‐}x^{T}\left(\Theta =90^{◦}\right)\equiv \mathrm{Mounting ‐}z^{T}\left(\Theta =90^{◦}\right)$$ 24

$$\mathrm{Mounting}\;y^{T}\left(\Theta =90^{◦}\right)\equiv \mathrm{Mounting ‐}z^{T}\left(\Theta =0^{◦}\right)$$ 25

where the mountings are as shown in Figure 2c. This pairwise coincidence arises as the NMR interactions are invariant under rotation about the magnetic field axis.^52^

We attempted to observe any ^11^B satellite transitions $\left(\left|\frac{3}{2}\right\rangle ↔\left|\frac{1}{2}\right\rangle ,\left|-\frac{1}{2}\right\rangle ↔\left|-\frac{3}{2}\right\rangle \right)$ over ± 519 ppm/100 kHz (see the Supporting Information) for a limited number of orientations but any satellite peaks were below our detection limit. As shown in Figure 7, our simulation for the ^11^B single-crystal NMR spectra suggests that the satellite transitions should be found in a ± 1.5 MHz window. We can remove the effect of the first-order quadrupole by adding the satellite frequencies, as described in ref.^53^ The resulting contributions of the second-order quadrupolar–CSA and quadrupolar–ACS couplings are also shown in Figure 7—these are on the order of Hz. Additional magnet time will be required to more exhaustively measure the likely broad and weak satellites experimentally.

### Analysis of Single-Crystal NMR Spectra

#### Quadrupolar and Chemical Shielding Tensors

The optimized quadrupolar and CSA parameters, along with their errors, were obtained by fitting the middle transition of each site using the Analysis of Single-Crystal Spectra (ASICS) software package.^52^ Since the coupling partner for ^11^B is ^14^N, the *m* = 0 energy level of ^14^N does not influence the middle transition of ^11^B.

The rotation plots shown in Figures 8 and 9 were fit according to eqs 11 and 12. Tables 5 and 6 summarize the optimized coefficients provided by ASICS for the magnetically nonequivalent B nuclei in (-,-)-8-HQ­(ipc)_2_B and (+,+)-8-HQ­(ipc)_2_B, respectively.

**Figure 8:**
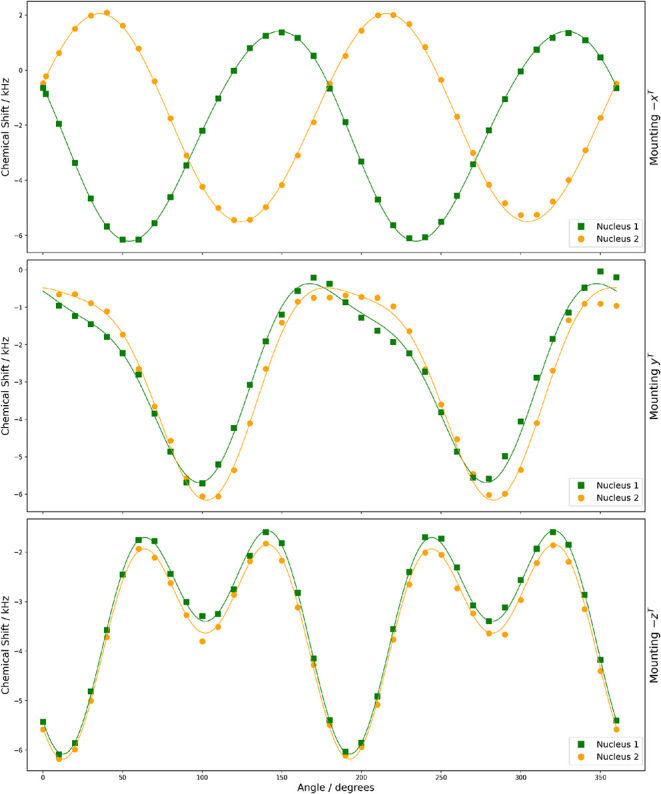
Rotation plots for the ^11^B central transition in (-,-)-8-HQ­(ipc)_2_B showing experimental resonances with two magnetically nonequivalent ^11^B nuclei under rotation of the three orientations of the crystal sample. The curves are constructed from optimized coefficients (see Table 5).

**Figure 9:**
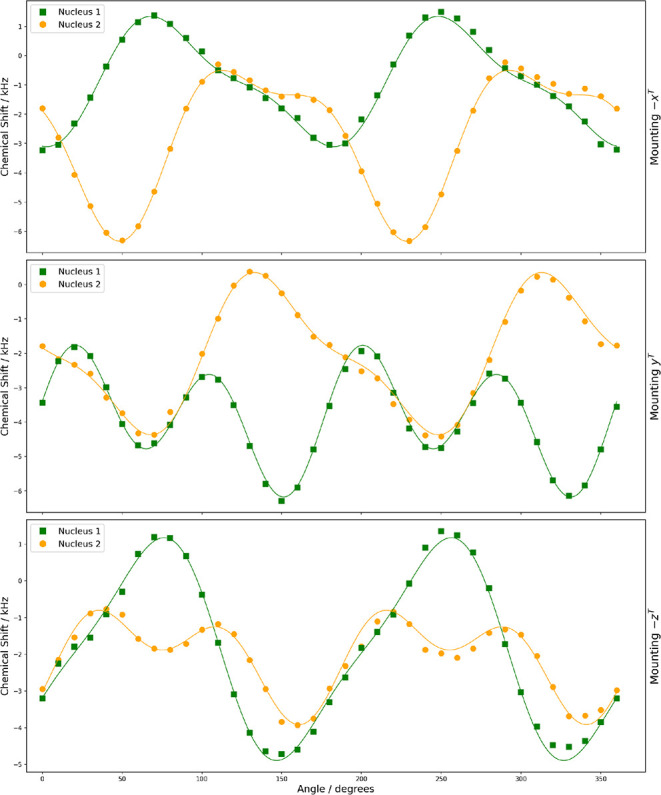
Rotation plots for the ^11^B central transition in (+,+)-8-HQ­(ipc)_2_B showing experimental resonances with two magnetically nonequivalent ^11^B nuclei under rotation of the three orientations of the crystal sample. The curves are constructed from optimized coefficients (see Table 6).

Table 7 summarizes the optimized experimental parameters, with error limits estimated as 95% confidence intervals of individual parameters for the ^11^B nucleus, along with the DFT calculations of quadrupolar coupling and CSA parameters for ^11^B and ^14^N nuclei in both the enantiomers of 8-HQ­(ipc)_2_B. For the ^11^B nucleus, we observed good correspondence between the theoretically computed and the experimental parameters, within 95% confidence intervals. For some parameters, the confidence interval could not be reliably estimated as chi-square values (goodness of fit) for those parameters were not normally distributed.

Enantiomers are expected to have the same quadrupolar coupling tensor, CSA tensor, and relative orientation of both tensors in the PAS. The experimentally determined parameters for both enantiomers are indeed similar. However, the calculated *C*_*Q*_ values are lower, and the η_*Q*_ values are higher, for both enantiomers when compared to the experimental values. The ^11^B NMR parameters obtained in this study are most comparable to those reported for boronic esters by Weiss et al.^54^ This correspondence is likely coincidental, as the boronic esters have a trigonal planar boron center attached to an aromatic ring and two oxygens, while the tetrahedral boron in this study’s 8-HQ­(ipc)_2_B molecule is attached to a phenolic oxygen, an aromatic nitrogen, and two aliphatic carbons, so the borons in the previous work and this study have quite dissimilar coordination and electronic environments.

The Euler angles (*a*, *b*, and *c*) describing PAF (CSA) relative to PAF­(Q) are also listed in Table 7. Among these, the angle *a* shows the largest statistical uncertainty, likely due to the inaccurate determination of η_CS_ which varies between 67% and 100% and has correlation with the Euler angle *a*.^55^ Because the projection direction of the quadrupolar and CSA tensor principal axes can be ambiguous, the Euler angles are rotated by 180^◦^ when necessary to enable direct comparison. The triple of Euler angles relating the quadrupolar PAF with crystal axis frame were also calculated and are listed in the Supporting Information.

#### Spin–Spin Coupling

To qualitatively analyze the spin–spin coupling in the rotation plots for both enantiomers, a similar approach to that of Lumsden et al.^56^ was followed. The upper and lower bounds on the magnitude of the ^11^B–^14^N spin–spin coupling (Δν) as the sample is systematically rotated about the magnetic field are given as

26 $$\Delta \nu_{\max}=J_{\mathrm{iso}}-2\left(R_{\mathrm{DD}}-\Delta J/3\right)$$

27 $$\Delta \nu_{\min}=J_{\mathrm{iso}}+\left(R_{\mathrm{DD}}-\Delta J/3\right)$$

where *J*_iso_ is the isotropic indirect spin–spin coupling constant, Δ*J* is the anisotropy of the indirect spin–spin coupling tensor, and *R*_DD_ is the magnitude of direct dipolar coupling between the ^11^B and ^14^N nuclei given as

28 $$R_{\mathrm{DD}}=-\left(\frac{\mu_{0}}{4\pi }\right)\left(\frac{\hbar }{2\pi }\right)\left(\frac{\gamma_{11_{B}}\gamma_{14_{N}}}{\left\langle r_{\mathrm{BN}}^{3}\right\rangle }\right)$$

*J*_iso_ was calculated to be 6.0 Hz and *R*_DD_ to be −679.1 Hz (*r*_BN_ = 1.6 Å) for both the enantiomers using DFT calculations. Figures 10 and 11 show the variation of the spin–spin dipolar coupling between ^11^B–^14^N for rotation about the three orthogonal tenon axes, along with the DFT-calculated range for spin–spin dipolar coupling. As the magnitudes of the values Δν rarely exceed Δ ν_max_ and Δ ν_min_, our results suggest little contribution from the anisotropic part of the ***J*** tensor.

**Figure 10:**
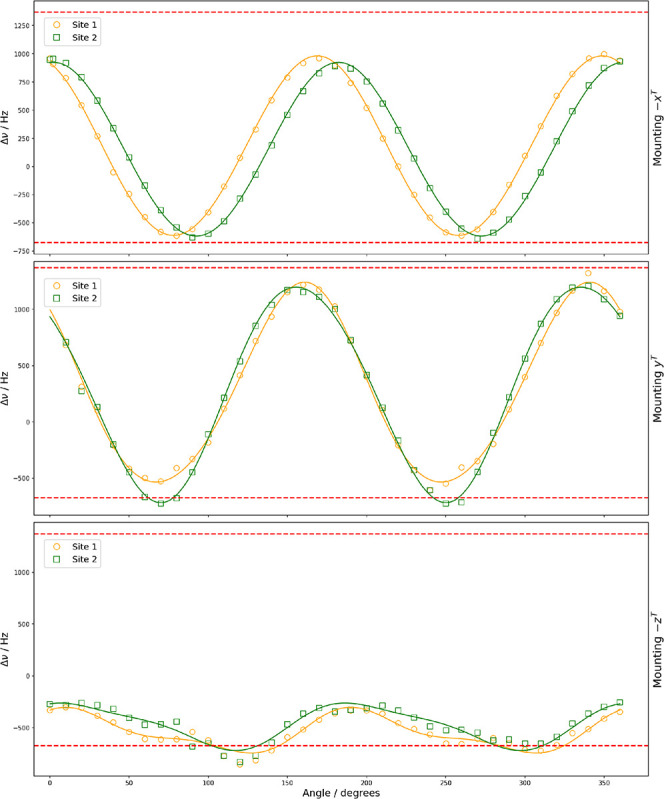
Rotation plots for the ^11^B–^14^N spin–spin coupling in (-,-)-8-HQ­(ipc)_2_B showing experimental resonances with two magnetically nonequivalent sites under rotation of the three orientations of the crystal sample. The dashed line show calculated range of the spin–spin coupling from the DFT calculations.

**Figure 11:**
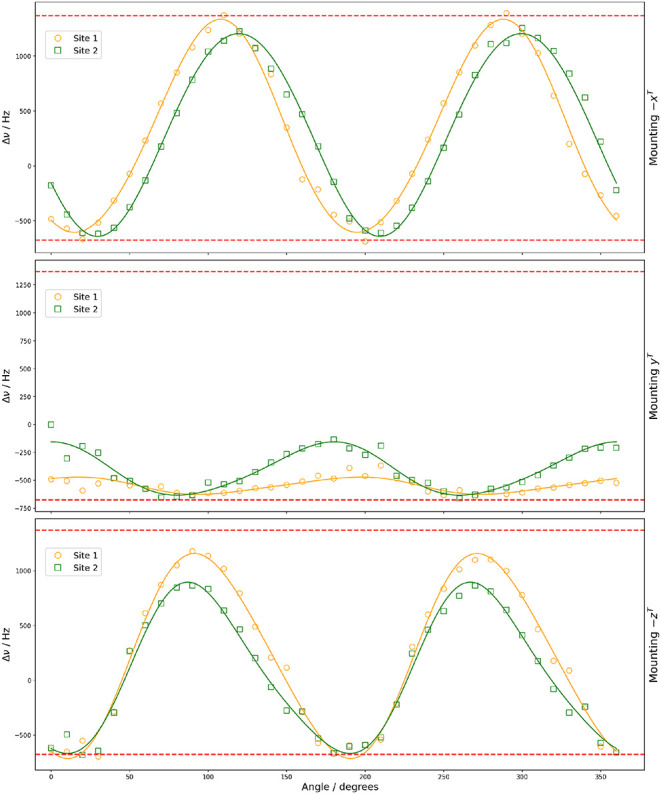
Rotation plots for the ^11^B– ^14^N spin–spin coupling in (+,+)-8-HQ­(ipc)_2_B showing experimental resonances with two magnetically nonequivalent sites under rotation of the three orientations of the crystal sample. The dashed line shows the calculated range of the spin–spin coupling from the DFT calculations.

## Conclusions

We have investigated the ^11^B sites in a pair of chiral enantiomers using single-crystal NMR spectroscopy to probe both quadrupolar and chemical shielding tensor components, thereby providing insights into the magnetic and electronic structures of chiral systems. By analyzing the central transition $\left(\left|\frac{1}{2}\right\rangle ↔\left|-\frac{1}{2}\right\rangle \right)$ of ^11^B, we determined the NMR quadrupolar tensor parameters for both enantiomers of 8-HQ­(ipc)_2_B. These parameters were found to be statistically similar within a 95% confidence interval, as expected, underscoring the utility of single-crystal NMR in resolving NMR tensor components in chiral compounds. The good agreement between the experimental results and DFT calculations further supports the validity of our findings.

However, the ^14^N peak transitions and satellite transitions for ^11^B were not observable. For ^14^N, this is attributed to its low gyromagnetic ratio, while for ^11^B, the absence of satellite transitions is likely due to the limited number of scans and potential crystal imperfections that broaden the satellite lines more severely than the central transition. These limitations hinder the experimental detection of quadrupolar-antisymmetric chemical shift (Q-ACS) cross-terms, which are only accessible through nonsymmetric satellite transitions such as $\left|\frac{3}{2}\right\rangle ↔\left|\frac{1}{2}\right\rangle$ and $\left|-\frac{1}{2}\right\rangle ↔\left|-\frac{3}{2}\right\rangle$ . In symmetric transitions like the central transition, the Q-ACS cross-terms vanish and thus are unobservable.

Despite these experimental constraints, DFT-calculated antisymmetric components of the chemical shielding tensor (ACS) for each enantiomer exhibit the expected chiral signatures—namely, equal magnitudes with opposite signs for corresponding components except for the component lying in the mirror symmetry plane. For ^11^B, the ACS components are on average approximately 3% the magnitude of the anisotropic chemical shift, suggesting that chiral influences would manifest as Δ*δ*∼± 4–6 ppm, provided that Q-ACS interactions in the satellite transitions could be observed in future studies.

DFT calculations yield valuable information about the extended electronic structure of the chiral environment. Although the nitrogen atom in 8-HQ­(ipc)_2_B is not itself a stereogenic chiral center—it lies within a mirror plane and adopts a coplanar *sp* ^2^-hybridized geometry—it nonetheless participates in the chiral framework of the molecule. As a result, its chemical shift tensor also contains antisymmetric components, which appropriately invert in sign between the enantiomers. The magnitude of the ACS components for ^14^N is approximately 17% of the anisotropic chemical shift.

This study underscores the need for further experimental investigations to access quadrupolar-antisymmetric chemical shift (Q-ACS) cross-terms through satellite transitions of quadrupolar nuclei in chiral systems. Such studies would greatly enhance the applicability of solid-state NMR spectroscopy for probing the molecular chirality. Future work will focus on exploring cross-correlations between quadrupolar and chemical shielding tensors in quadrupolar nuclei, aiming to deepen our understanding of chiral electronic environments and broaden the impact of NMR in areas such as materials science, pharmaceutical development, stereochemical analysis, and astrobiology.

## Supplementary Material





## Notes

X-ray orientation of (+,+) and (-,-)-8-HQ­(ipc)­2B, in-house deconvolution program script, NMR pulse program used for data collection, NMR wide scan spectrum, Euler angles relating the PAF­(Q) to goniometer and crystal axis frame (PDF)
NMR spectra (XLSX)
