# Supplementary material for: Probing the 11B Quadrupolar and Chemical Shielding Tensors in a Pair of Organoboron Enantiomers
Source: J Phys Chem A. 2025 Nov 11;129(46):10601–15. doi: 10.1021/acs.jpca.5c03645 (PMC12641489; doi:10.1021/acs.jpca.5c03645)
Supplement: Supplementary file 1 [file jp5c03645_si_001.pdf]

Supporting Information

Probing the ^{11}B Quadrupolar and Chemical Shielding Tensors in a Pair of Organoboron Enantiomers

Shiva Agarwal[1], Zhongrui Li[2], Jason Kitchen[3], Sungsool Wi[3]*, and John B. Miller[4]†

[1]Department of Physics, Western Michigan University, Kalamazoo, MI 49008, USA
[2]Electron Microbeam Analysis Laboratory, University of Michigan, Ann Arbor, MI, 48109, USA
[3]National High Magnetic Field Laboratory, Tallahassee, FL, 32310, USA
[4]Department of Chemistry, Western Michigan University, Kalamazoo, MI 49008, USA

*Corresponding author: sungsool@magnet.fsu.edu
†Corresponding author: john.b.miller@wmich.edu

1 Calculation of Orientation Angles

From x-ray diffraction (XRD), the orientation of a single crystal with respect to the tenon on which it was mounted, were obtained. The XRD procedure marked the surface normal direction and an edge plane of the crystal. This information was used to find the other two orthogonal axes of the tenon in terms of Miller indices (*hkl*) of the unit cell in the sample. The direction cosines of the crystal axes ($a,\ b,\ c$) with respect to the tenon axes ($X,\ Y,\ Z$) were then specified.

1.1 Orientation of (+,+)-8-HQ$(ipc)_2$B

Since 8-HQ$(ipc)_2$B crystal system is monoclinic, the oblique system of the crystal axes was transformed into an orthogonal axes set so that the Euler angles (α, β, γ) relating the crystal axes to the tenon frame could be determined. The orthogonalization of axes and determination of Euler angles were performed using TRAFO [1]. From XRD the surface normal of (+,+)-8-HQ$(ipc)_2$B was determined to be in the direction (1 0 -2) and the edge plane was oriented toward (0 1 0). Figure S1 shows the relevant XRD plots and Figure S2 shows surface normal direction and edge plane for (+,+)-8-HQ$(ipc)_2$B crystal sample.

A fiduciary dot on the tenon was marked so that the X direction of the plate could be fixed. The Y axis of the plate is perpendicular to this direction. The angle between (0 1 0) and the tenon Y direction was found to be $27.0° \pm 0.5°$ (see Figure S3).

Find Y direction as crystallographic vector

Let $(y_1\ y_2\ y_3)$ be a unit vector making an angle of 100° with (0 1 0) vector. The dot product of both vectors gives:

$$(0\ 1\ 0)\cdot(y_1\ y_2\ y_3) = cos\ 27°$$
$$y_2 \approx 0.9$$

The surface normal or **Z** vector is perpendicular to **Y** vector

$$\implies \mathbf{Z}\cdot\mathbf{Y} = 0$$
$$(1\ 0\ -2)\cdot(y_1\ y_2\ y_3) = 0$$
$$y_1 = 2y_3$$

(a)

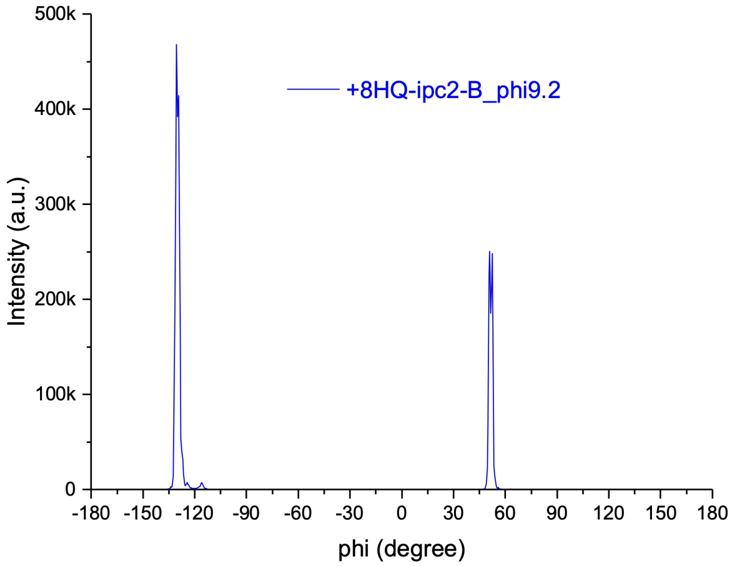

(b)

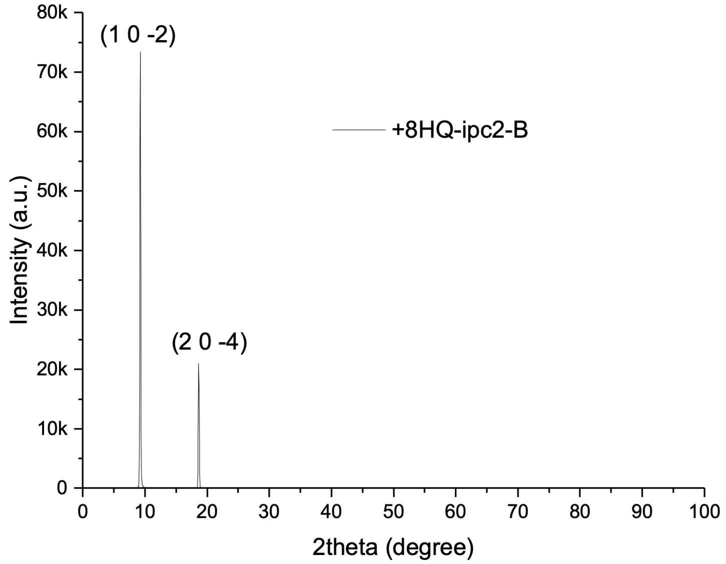

Figure S1: Experimental x-ray diffraction patterns showing **(a)** Intensity vs. ϕ and **(b)** Intensity vs. 2θ plots for the (+,+)-8-HQ(ipc)$_2$B single crystal.

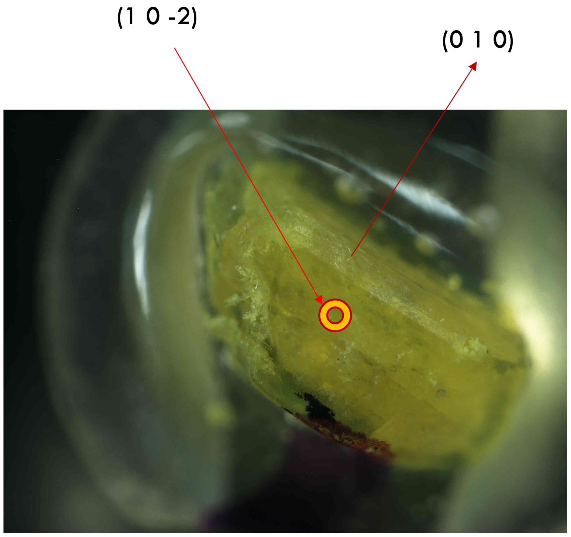

Figure S2: The circle in the picture marks the surface normal direction (1 0 -2) and the red arrow shows the edge plane (0 1 0) of the (+,+)-8-HQ(ipc)$_2$B single crystal.

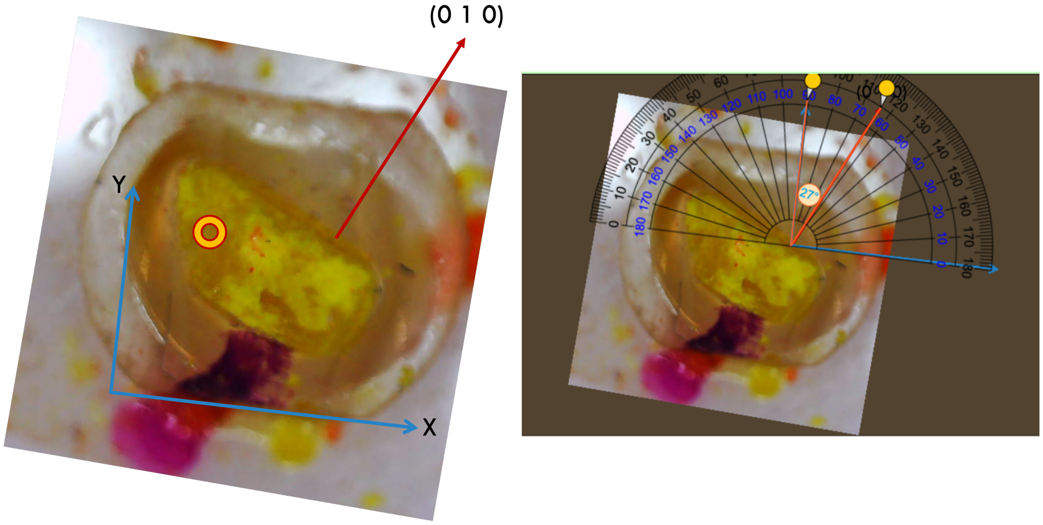

Figure S3: *Left:* Top view of the crystal showing tenon with the glued crystal. A mark can be seen at the bottom of the plate. The mark was used as a guide to set the X and Y direction of the plate. *Right:* Measurement of angle between (0 1 0) and Y vector was done using online protractor tool [2]

Since **Y** must be a unit vector

$$\begin{aligned} y_1^2 + y_2^2 + y_3^2 &= 1 \\ (2y_3)^2 + 0.9^2 + y_3^2 &= 1 \\ y_3 &= \pm 0.2 \\ \text{taking } y_3 &= 0.2 \\ y_1 &= 0.4 \\ \mathbf{Y} = (0.4\ 0.9\ 0.2) \end{aligned}$$

In terms of crystallographic directions,

$$\mathbf{Y} = (4\ 9\ 2)$$

The angle θ between (0 1 0) and **Y** can be verified as

$$\begin{aligned} (0\ 1\ 0) \cdot \mathbf{Y} &= |(0\ 1\ 0)||\mathbf{Y}|\ cos\ \theta \\ \theta &= cos^{-1}(9/\sqrt{101}) \\ &= 26.4^\circ \end{aligned}$$

Find X direction as crystallographic vector

Since $\mathbf{X}, \mathbf{Y}, \mathbf{Z}$ vectors are orthogonal to each other

$$\begin{aligned} \mathbf{X} &= \mathbf{Y} \times \mathbf{Z} \\ &= (4\ 9\ 2) \times (1\ 0\ -2) \\ &= (-18\ 10\ -9) \end{aligned}$$

The angle ϕ between (0 1 0) and **X** can be verified as

$$\begin{aligned} (0\ 1\ 0) \cdot \mathbf{X} &= |(0\ 1\ 0)||\mathbf{X}|\ cos\ \phi \\ \phi &= cos^{-1}(10/\sqrt{505}) \\ &= 63.6^\circ \end{aligned}$$

The crystal axes and the tenon directions on the unit cell of (+,+)-8-HQ(ipc)$_2$B were visualized in *VESTA* [3] (see Figure S4) to verify that they represent the measured crystal axes and the physical plate directions.

The direction cosines of the crystal axes (*a, b, c*) with respect to the tenon axes (*X, Y, Z*) were calculated to be

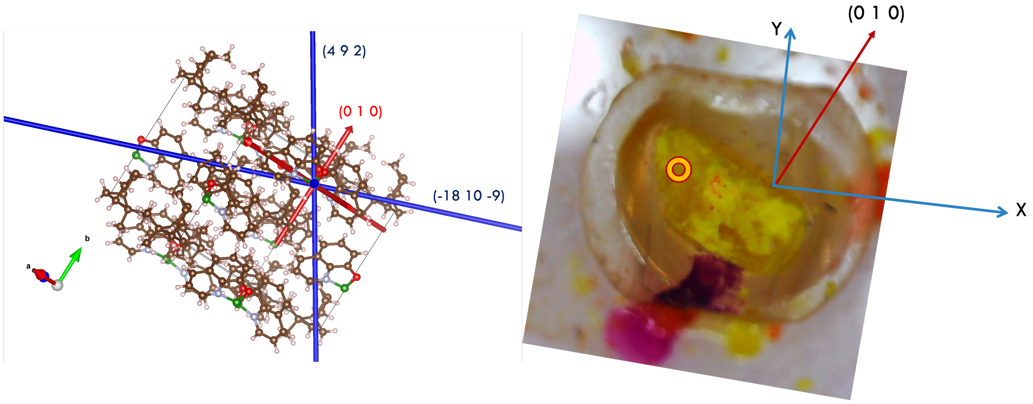

Figure S4: Crystal axes represented in red and tenon axes represented in blue color. *Left:* Representation of axes in the unit cell of (+,+)-8-HQ(ipc)$_2$B *Right:* Representation of axes on the single crystal of (+,+)-8-HQ(ipc)$_2$B

	X axis	Y axis	Z axis
a axis	$\frac{-18}{\sqrt{505}}$	$\frac{4}{\sqrt{101}}$	$\frac{1}{\sqrt{5}}$
b axis	$\frac{10}{\sqrt{505}}$	$\frac{9}{\sqrt{101}}$	$\frac{0}{\sqrt{5}}$
c axis	$\frac{-9}{\sqrt{505}}$	$\frac{2}{\sqrt{101}}$	$\frac{-2}{\sqrt{5}}$

The fractional coordinates of the oblique axis system calculate as direction cosines of crystal axis with respect to tenon axes were orthogonalized to Cartesian coordinates using TRAFO software. TRAFO was also used to calculate Euler angles from the direction cosines of the orthogonalized crystal axis system in the tenon frame. The triple of Euler angles (α, β, γ) retrieved that specify the orientation of both systems was found to be $153.5°, 153.4°, 179.9°$ (see Figure S5).

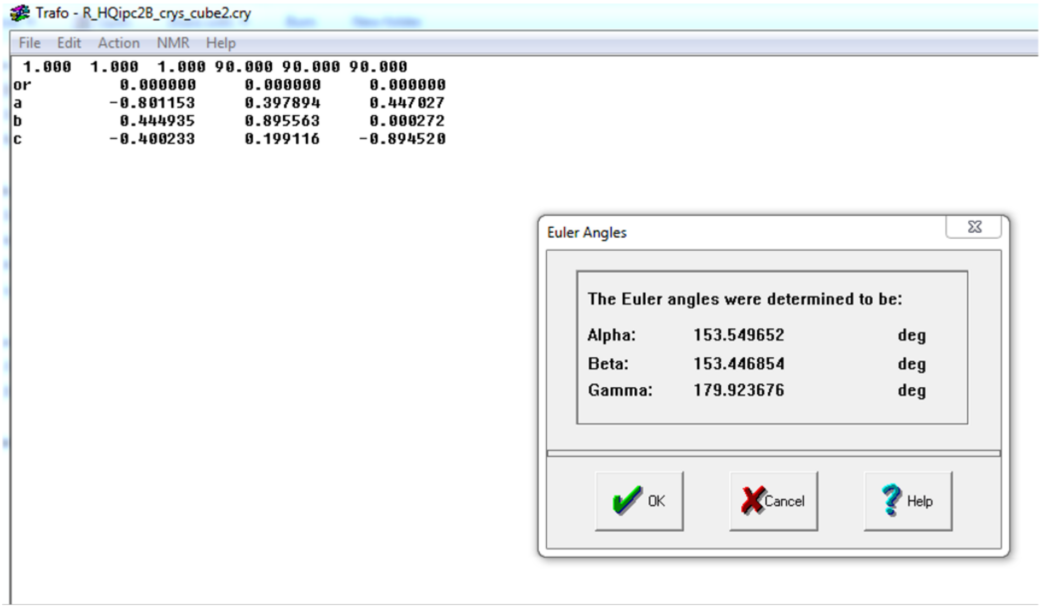

Figure S5: Calculation of orientation angles for (+,+)-8-HQ(ipc)$_2$B in TRAFO

For transformation from frame A with basis $\{X_A, Y_A, Z_A\}$ to frame B with basis $\{X_B, Y_B, Z_B\}$, passive rotation by an angle α around the Z_A axis is performed to result in an intermediate frame $\{X'_A, Y'_A, Z_A\}$. The first rotation is followed by a passive rotation by an angle β around the new Y'_A axis that results in frame $\{X''_A, Y'_A, Z_B\}$. Finally, passive rotation around Z_B by an angle γ is carried out. Figure S6 shows the step-wise rotations as described. All rotations are counter clockwise.

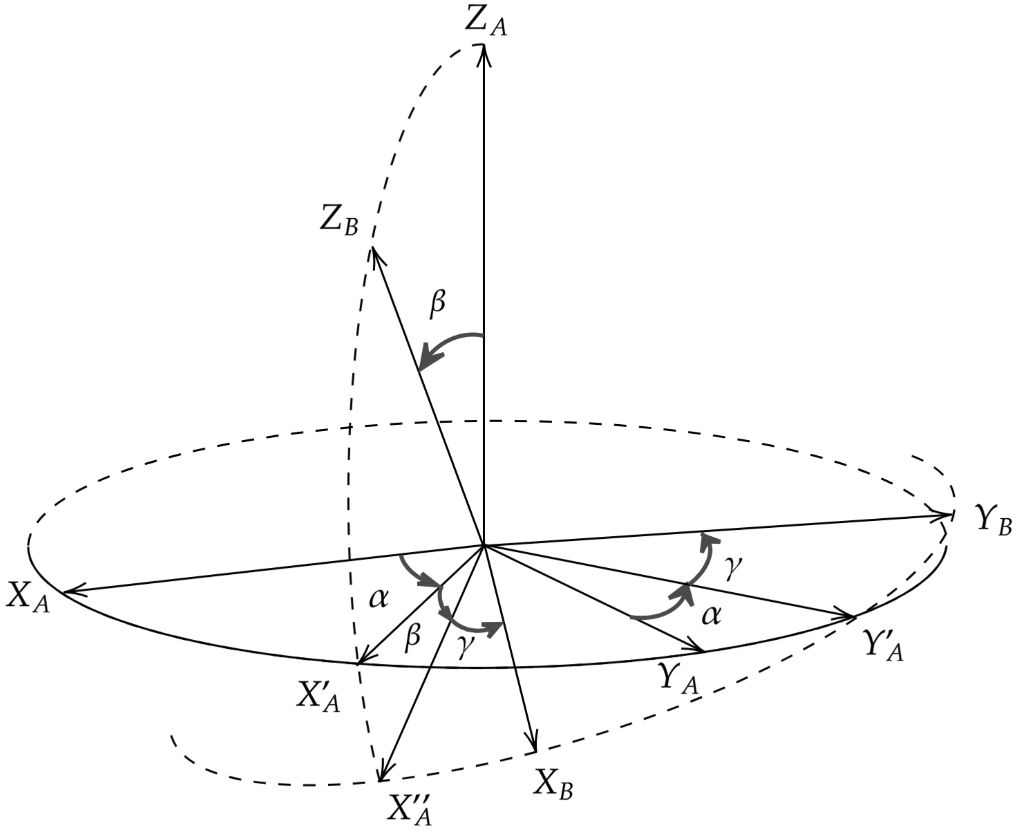

Figure S6: Transformation of frame A to frame B using rotations defined by the Euler triplet (α, β, γ).

1.2 Orientation of (-,-)-8-HQ(ipc)$_2$B

From XRD the surface normal of (-,-)-8-HQ(ipc)$_2$B was determined to be in the direction (1 0 1) and the edge plane was oriented toward (0 1 0). Figure S7 shows the relevant XRD plots and Figure S8 shows surface normal direction and edge plane for the (-,-)-8-HQ(ipc)$_2$B crystal sample. The angle between (0 1 0) and tenon Y direction was found to be $100.0° \pm 0.5°$ (see Figure S9).

(a)

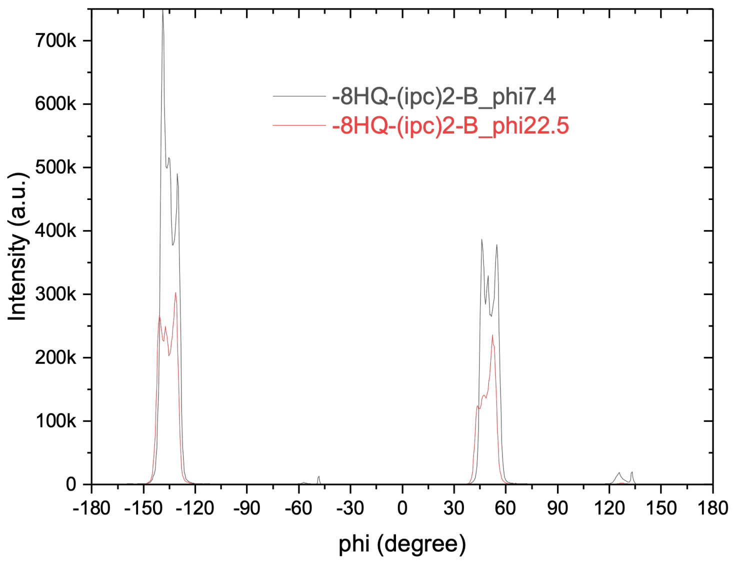

(b)

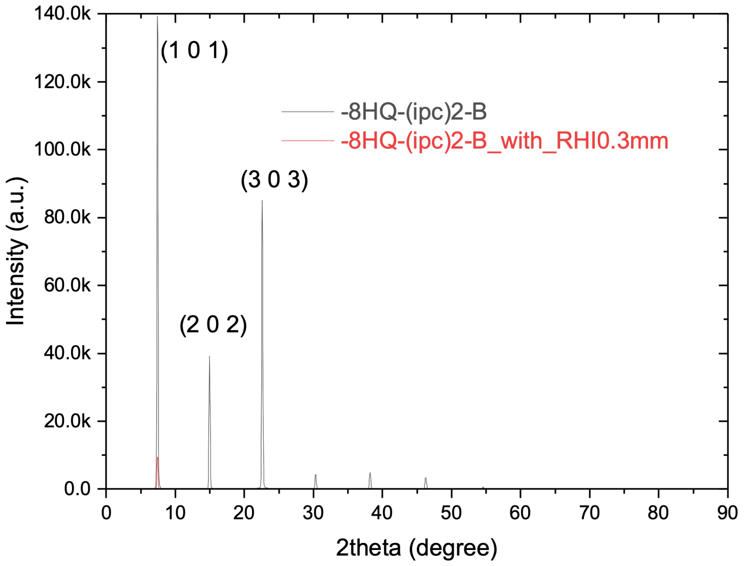

Figure S7: Experimental x-ray diffraction patterns showing **(a)** Intensity vs. ϕ and **(b)** Intensity vs. 2θ plots for (-,-)-8-HQ(ipc)$_2$B single crystal.

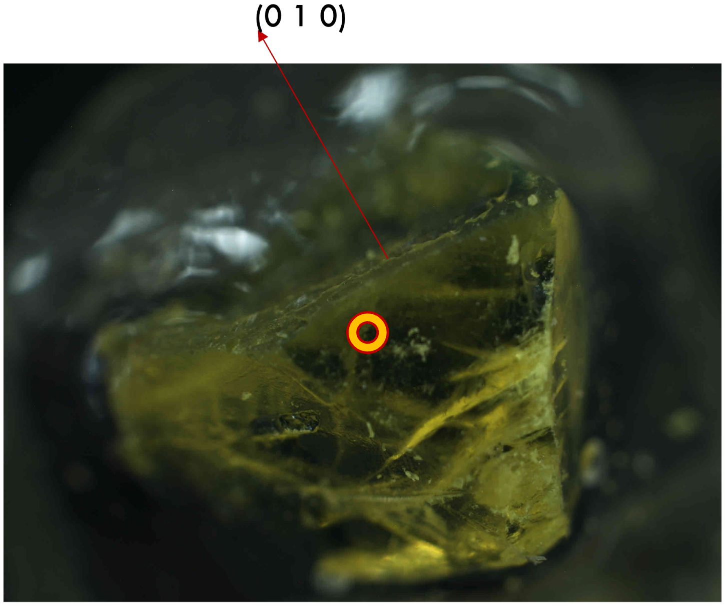

Figure S8: The circle in the picture marks the surface normal direction (1 0 1) and the red arrow shows the edge plane (0 1 0) of the (-,-)-8-HQ(ipc)$_2$B single crystal.

Find Y direction as crystallographic vector

Let $(y_1\ y_2\ y_3)$ be a unit vector making an angle of 100° with (0 1 0) vector. The dot product of both vectors gives:

$$\begin{aligned}(0\ 1\ 0)\cdot(y_1\ y_2\ y_3) &= cos\ 100^\circ \\ y_2 &\approx -0.174\end{aligned}$$

The surface normal or **Z** vector is perpendicular to **Y** vector

$$\begin{aligned}\implies \mathbf{Z}\cdot\mathbf{Y} &= 0 \\ (1\ 0\ 1)\cdot(y_1\ y_2\ y_3) &= 0 \\ y_1 &= -y_3\end{aligned}$$

Since **Y** must be a unit vector

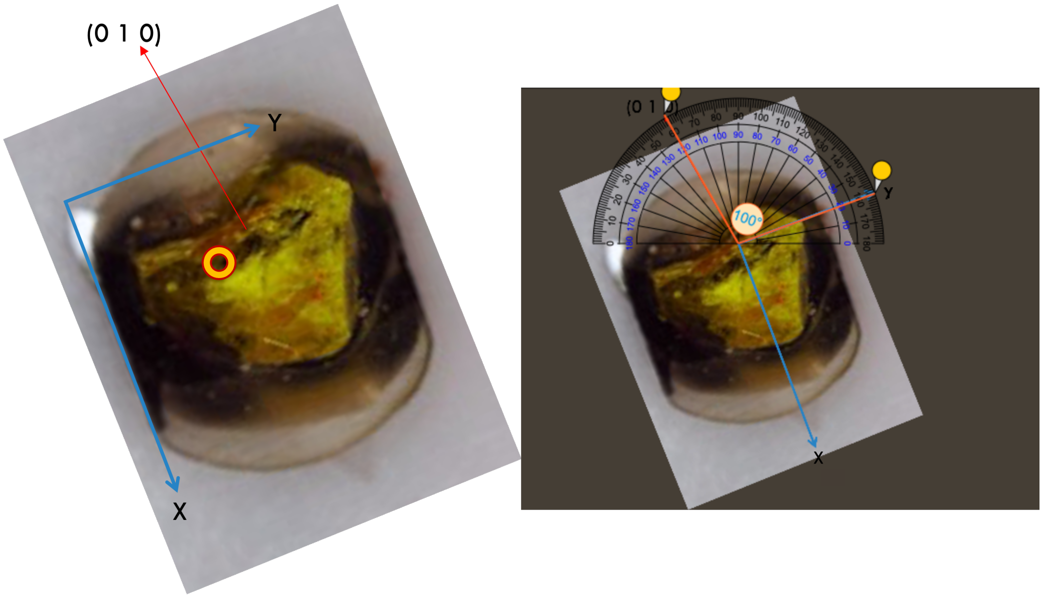

Figure S9: *Left:* Top view of the crystal showing tenon with the glued crystal. A mark representing X direction of the plate can be seen at the left hand side. The mark was used as a guide to set the X and Y direction of the plate. *Right:* Measurement of angle between (0 1 0) and Y vector using online protractor tool.

$$\begin{aligned} y_1^2 + y_2^2 + y_3^2 &= 1 \\ (-y_3)^2 + (-0.174)^2 + y_3^2 &= 1 \\ y_3 &= \pm 0.696 \\ \text{taking } y_3 &= -0.696 \\ y_1 &= 0.696 \\ \mathbf{Y} = (0.696 \;\; -0.174 \;\; -0.696) \end{aligned}$$

In terms of crystallographic directions,

$$\mathbf{Y} = (4 \;\; -1 \;\; -4)$$

The angle θ between (0 1 0) and $\mathbf{Y}$ can be verified as

$$\begin{aligned}(0\ 1\ 0)\cdot\mathbf{Y} &= |(0\ 1\ 0)||\mathbf{Y}|\ cos\ \theta \\ \theta &= cos^{-1}(-1/\sqrt{33}) \\ &= 100.0^{\circ}\end{aligned}$$

Find X direction as crystallographic vector

Since $\mathbf{X}, \mathbf{Y}, \mathbf{Z}$ vectors are orthogonal to each other

$$\begin{aligned}\mathbf{X} &= \mathbf{Y}\times\mathbf{Z} \\ &= (4\ \ -1\ \ -4)\times(1\ 0\ 1) \\ &= (-1\ \ -8\ 1)\end{aligned}$$

The angle ϕ between (0 1 0) and $\mathbf{X}$ can be verified as

$$\begin{aligned}(0\ 1\ 0)\cdot\mathbf{X} &= |(0\ 1\ 0)||\mathbf{X}|\ cos\ \phi \\ \phi &= cos^{-1}(-8/\sqrt{66}) \\ &= 170^{\circ}(\text{or} - 190^{\circ})\end{aligned}$$

The calculated crystal and tenon axes agreed well with the measured axes (see Figure S10).

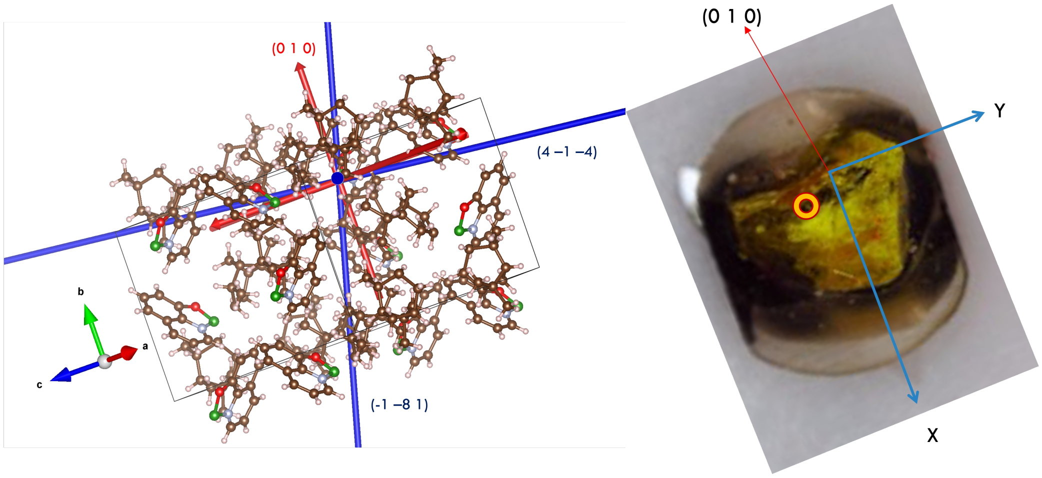

Figure S10: Crystal axes represented in red and tenon axes represented in blue color. *Left:* Representation of axes in the unit cell of (-,-)-8-HQ(ipc)$_2$B
.
Right: Representation of axes on the single crystal of (-,-)-8-HQ(ipc)$_2$B

The direction cosines of the crystal axes (a, b, c) with respect to the tenon axes (X, Y, Z) were calculated to be

	X axis	Y axis	Z axis
a axis	$\frac{-1}{\sqrt{66}}$	$\frac{4}{\sqrt{33}}$	$\frac{1}{\sqrt{2}}$
b axis	$\frac{-8}{\sqrt{66}}$	$\frac{-1}{\sqrt{33}}$	$\frac{0}{\sqrt{2}}$
c axis	$\frac{1}{\sqrt{66}}$	$\frac{-4}{\sqrt{33}}$	$\frac{1}{\sqrt{2}}$

Using TRAFO, the triple of Euler angles that specify the orientation of the orthogonalized crystal axis system in the tenon frame was found to be $280.0°, 45.0°, 180.0°$ (see Figure S11).

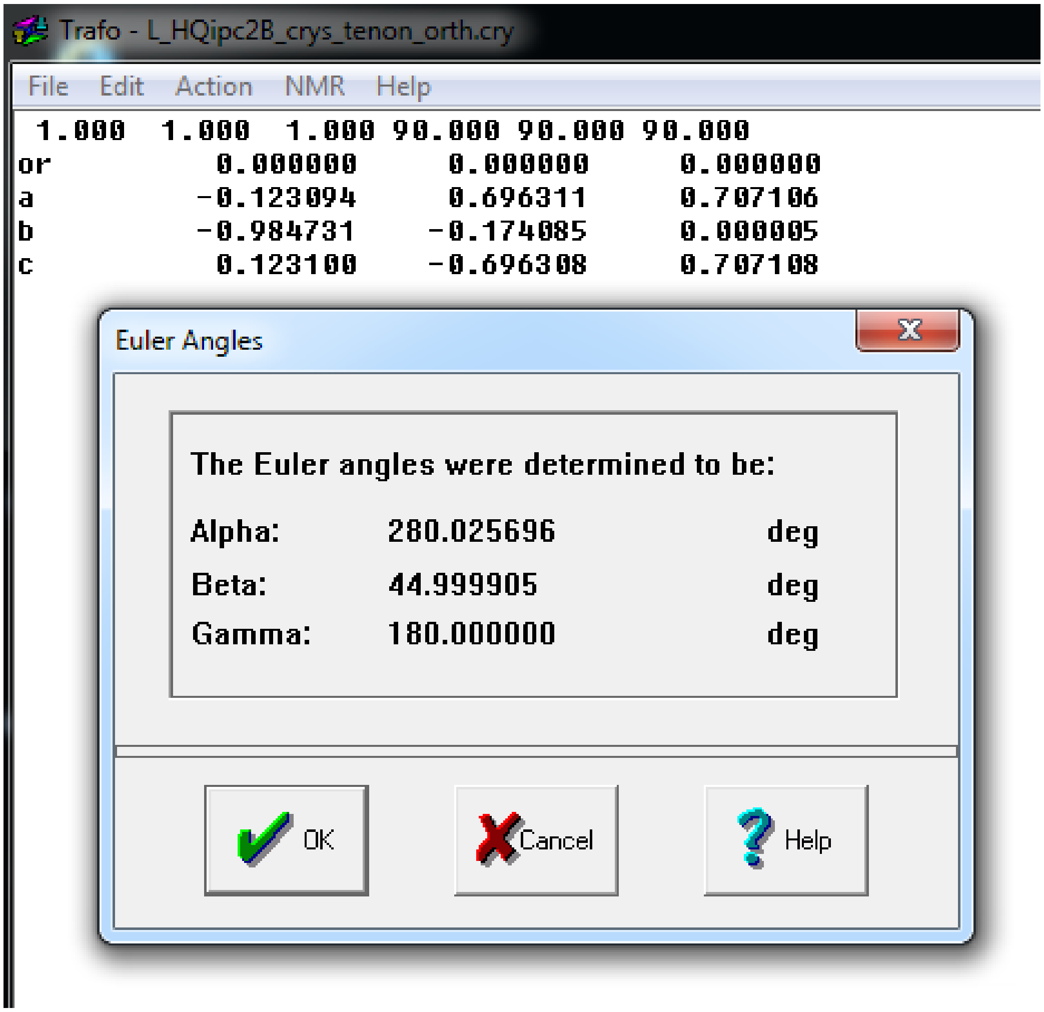

Figure S11: Calculation of orientation angles for (-,-)-8-HQ(ipc)$_2$B in TRAFO

2 Code Listings

Following code is also available on GitHub

```
import time
import csv
import numpy as np
import matplotlib.pyplot as plt

# everything in iminuit is done through the Minuit object, so
     we import it
from iminuit import Minuit

# we also need a cost function to fit and import the
   LeastSquares function
from iminuit.cost import LeastSquares

# display iminuit version
import iminuit
from jacobi import propagate
print("iminuit version:", iminuit.__version__)

def lorentz(x, gamma, x0, A): #define lorentz fnc for single
   peak
    return (A*gamma/(np.pi*((x - x0)**2) + gamma**2))

def lorentz_cluster_singlet(x, gamma, center_1,  center_2,
   center_3, center_4, center_5, center_6, A, offset): #
   define fnc for cluster of six individual peaks
        return lorentz(x, gamma, center_1, A) + lorentz(x,
   gamma, center_2, A)  + lorentz(x, gamma, center_3, A) +
   lorentz(x, gamma, center_4, A) + lorentz(x, gamma,
   center_5, A) + lorentz(x, gamma, center_6, A) + offset

path = "/home/shiva/WMU/PhD/Scripts/phd_project/Python/NMR/
   Data_exp/" #path for data file

#value from data file
steps = 32768
x_start = 100.06
x_end = -106.0
steps_ppm = (x_end - x_start)/steps
x = np.arange(x_start, x_end, steps_ppm)

#Input for angle in simulation
```

```
file_start = 96                                    #enter the
   first file number
file_end = 133                                     #enter the last
    file number
skip_file = []

angle_start = 0
angle_end = 360
angle_step = 10

xleft = 20                                         # left x limit
   on plot
xright = -40                                       # right x limit
    on plot

timestamp = time.strftime("%Y%m%d_%H%M%S")   #timestamp to
   seperate files
out_file = f"fit_data_{str(file_start)}_{str(file_end)}_{
   timestamp}.csv"  #outfile name

#********************************-8HQ X rotation
   ********************************
#****************provide initial values
   ******************************************
initializer_list = [
    {'gamma': 1, 'center_1': -3, 'split_1': 5, 'center_2':
   -3, 'split_2': 5, 'A': 1, 'offset': 0.}, #96
    {'gamma': 1, 'center_1': 1, 'split_1': 3, 'center_2': -5,
    'split_2' : 3, 'A': 1, 'offset': 0.}, #97
    {'gamma': 1, 'center_1': 3, 'split_1': 5, 'center_2':
   -10, 'split_2' : 5, 'A': 1, 'offset': 0.}, #98
    {'gamma': 1, 'center_1': 8, 'split_1': 1, 'center_2':
   -18, 'split_2' : 1, 'A': 1, 'offset': 0.}, #99
    {'gamma': 1, 'center_1': 12, 'split_1': 1, 'center_2':
   -13, 'split_2': 1, 'A': 1, 'offset': 0.}, #100
    {'gamma': 1, 'center_1': 10, 'split_1': 1, 'center_2':
   -30, 'split_2' : 1, 'A': 1, 'offset': 0.}, #101
    {'gamma': 1, 'center_1': 9, 'split_1': 1, 'center_2':
   -32, 'split_2' : 1, 'A': 1, 'offset': 0.}, #102
    {'gamma': 1, 'center_1': 4, 'split_1': 1, 'center_2':
   -31, 'split_2' : 1, 'A': 1, 'offset': 0.}, #103
    {'gamma': 1, 'center_1': -1, 'split_1': 1, 'center_2':
   -31, 'split_2' : 1, 'A': 1, 'offset': 0.}, #104
    {'gamma': 1, 'center_1': -9, 'split_1': 1, 'center_2':
   -24, 'split_2' : 1, 'A': 1, 'offset': 0.}, #105
```

```
 {'gamma': 1, 'center_1': -16, 'split_1': 1, 'center_2':
-18, 'split_2' : 1, 'A': 1, 'offset': 0.}, #106
 {'gamma': 1, 'center_1': -22, 'split_1': 1, 'center_2':
-12, 'split_2' : 1, 'A': 1, 'offset': 0.}, #107
 {'gamma': 1, 'center_1': -26, 'split_1': 1, 'center_2':
-6, 'split_2' : 1, 'A': 1, 'offset': 0.}, #108
 {'gamma': 1, 'center_1': -30, 'split_1': 1, 'center_2':
-1, 'split_2' : 1, 'A': 1, 'offset': 0.}, #109
 {'gamma': 1, 'center_1': -28, 'split_1': 1, 'center_2':
4, 'split_2' : 1, 'A': 1, 'offset': 0.}, #110
 {'gamma': 1, 'center_1': -26, 'split_1': 1, 'center_2':
8, 'split_2' : 1, 'A': 1, 'offset': 0.}, #111
 {'gamma': 1, 'center_1': -30, 'split_1': 1, 'center_2':
-1, 'split_2' : 1, 'A': 1, 'offset': 0.}, #112
 {'gamma': 1, 'center_1': -16, 'split_1': 1, 'center_2':
6, 'split_2' : 1, 'A': 1, 'offset': 0.}, #113
 {'gamma': 1, 'center_1': -10, 'split_1': 1, 'center_2':
4, 'split_2' : 1, 'A': 1, 'offset': 0.}, #114
 {'gamma': 1, 'center_1': -3, 'split_1': 5, 'center_2':
-3, 'split_2' : 5, 'A': 1, 'offset': 0.}, #115
 {'gamma': 1, 'center_1': 4, 'split_1': 1, 'center_2':
-10, 'split_2' : 1, 'A': 1, 'offset': 0.}, #116
 {'gamma': 1, 'center_1': 8, 'split_1': 1, 'center_2':
-17, 'split_2' : 1, 'A': 1, 'offset': 0.}, #117
 {'gamma': 1, 'center_1': 10, 'split_1': 1, 'center_2':
-24, 'split_2' : 1, 'A': 1, 'offset': 0.}, #118
 {'gamma': 1, 'center_1': 11, 'split_1': 1, 'center_2':
-29, 'split_2' : 1, 'A': 1, 'offset': 0.}, #119
 {'gamma': 1, 'center_1': 9, 'split_1': 1, 'center_2':
-31, 'split_2' : 1, 'A': 1, 'offset': 0.}, #120
 {'gamma': 1, 'center_1': 4, 'split_1': 1, 'center_2':
-32, 'split_2' : 1, 'A': 1, 'offset': 0.}, #121
 {'gamma': 1, 'center_1': -2, 'split_1': 1, 'center_2':
-29, 'split_2' : 1, 'A': 1, 'offset': 0.}, #122
 {'gamma': 1, 'center_1': -9, 'split_1': 1, 'center_2':
-24, 'split_2' : 1, 'A': 1, 'offset': 0.}, #123
 {'gamma': 1, 'center_1': -15, 'split_1': 1, 'center_2':
-18, 'split_2' : 1, 'A': 1, 'offset': 0.}, #124
 {'gamma': 1, 'center_1': -22, 'split_1': 1, 'center_2':
-12, 'split_2' : 1, 'A': 1, 'offset': 0.}, #125
 {'gamma': 1, 'center_1': -25, 'split_1': 1, 'center_2':
-6, 'split_2' : 1, 'A': 1, 'offset': 0.}, #126
 {'gamma': 1, 'center_1': -27, 'split_1': 1, 'center_2':
-1, 'split_2' : 1, 'A': 1, 'offset': 0.}, #127
```

```
    {'gamma': 1, 'center_1': -27, 'split_1': 1, 'center_2':
   3, 'split_2' : 1, 'A': 1, 'offset': 0.}, #128
    {'gamma': 1, 'center_1': -25, 'split_1': 1, 'center_2':
   6, 'split_2' : 1, 'A': 1, 'offset': 0.}, #129
    {'gamma': 1, 'center_1': -20, 'split_1': 1, 'center_2':
   7, 'split_2' : 1, 'A': 1, 'offset': 0.}, #130
    {'gamma': 1, 'center_1': -15, 'split_1': 1, 'center_2':
   6, 'split_2' : 1, 'A': 1, 'offset': 0.}, #131
    {'gamma': 1, 'center_1': -8, 'split_1': 1, 'center_2':
   -2, 'split_2' : 1, 'A': 1, 'offset': 0.}, #132
    {'gamma': 1, 'center_1': -3, 'split_1': 5, 'center_2':
   -3, 'split_2' : 5, 'A': 1, 'offset': 0.} #133
    ]

# Initialize the CSV file
with open(path + out_file, 'w', newline='') as csvfile:
    fieldnames = ['file', 'center_1', 'center_2', 'center_3',
    'center_4', 'center_5', 'center_6', 'Comment']
    writer = csv.DictWriter(csvfile, fieldnames=fieldnames)
    writer.writeheader()

    encountered_angles = {}  # Dictionary to track
   encountered angles

    angle_counter = 0        # Initialize angle counter

    for numb, initial_values in zip(range(file_start,
   file_end+1), initializer_list):
        if numb in skip_file:
            continue
        # Calculate angle based on file number
        angle = f"$\\theta$  = {angle_counter * angle_step}"

        # Check if angle has been encountered before
        if angle in encountered_angles:
            # Skip this file if the angle has been
   encountered before
            continue
        else:
            # Add the angle to the dictionary
            encountered_angles[angle] = numb
            angle_counter += 1  # Increment angle counter
        yData = np.loadtxt(path+"SW_Shiva"+str(numb)+".txt",
   unpack=True )                              # read in
        yData = yData / max(yData)            # normalize
```

```
     yData = yData - min(yData)          # remove any
vertical offset
     least_squares = LeastSquares(x, yData, 0.1,
lorentz_cluster)
     m = Minuit(least_squares, **initial_values).migrad()

     residual = yData - lorentz_cluster(x, *m.values)  #
finding residual

     m_fit_info = [f"$\\chi^2$/$n_\\mathrm{{dof}}$ = {m.
fval:.1f} / {m.ndof:.0f} = {m.fmin.reduced_chi2:.1f}"]

 #Program to fit individual peaks using initial values
from program using center and split parameters

     least_squares_singlet = LeastSquares(x, yData, 0.1,
lorentz_cluster_singlet)
     m_singlet = Minuit(least_squares_singlet, gamma = m.
values['gamma'], center_1= (m.values['center_1'] -  m.
values['split_1']), center_2 =  m.values['center_1'],
                    center_3 = (m.values['center_1'] + m.
values['split_1']), center_4  =  (m.values['center_2'] - m
.values['split_2']),center_5 = m.values['center_2'],
                         center_6 =  (m.values['center_2']
 + m.values['split_2']), A =  m.values['A'], offset =  m.
values['offset']).migrad()

     residual = yData - lorentz_cluster_singlet(x, *
m_singlet.values)  # finding residual
     row_dict = {'file': numb,'Comment': ''}       # Define
 row_dict inside the loop

     m_singlet_fit_info = [f"$\\chi^2$/$n_\\mathrm{{dof}}$
 = {m_singlet.fval:.1f} / {m_singlet.ndof:.0f} = {
m_singlet.fmin.reduced_chi2:.1f}"]
  #****************Plotting
***********************************************

     peak_1 = lorentz(x, m_singlet.values['gamma'],
m_singlet.values['center_1'],m_singlet.values['A']) +
m_singlet.values['offset']
     peak_2 = lorentz(x, m_singlet.values['gamma'],
m_singlet.values['center_2'],m_singlet.values['A']) +
m_singlet.values['offset']
```

```
    peak_3 = lorentz(x, m_singlet.values['gamma'],
m_singlet.values['center_3'],m_singlet.values['A']) +
m_singlet.values['offset']
    peak_4 = lorentz(x, m_singlet.values['gamma'],
m_singlet.values['center_4'],m_singlet.values['A']) +
m_singlet.values['offset']
    peak_5 = lorentz(x, m_singlet.values['gamma'],
m_singlet.values['center_5'],m_singlet.values['A']) +
m_singlet.values['offset']
    peak_6 = lorentz(x, m_singlet.values['gamma'],
m_singlet.values['center_6'],m_singlet.values['A']) +
m_singlet.values['offset']
    plt.figure(figsize=(20,3))

    plt.plot(x, yData, '--', label="exp")
    plt.plot(x, lorentz_cluster_singlet(x, *m_singlet.
values), label="fit", color = 'olive')
    plt.plot(x,peak_1 )
    plt.plot(x, peak_2)
    plt.plot(x, peak_3)
    plt.plot(x, peak_4)
    plt.plot(x, peak_5)
    plt.plot(x, peak_6)
    plt.xticks(np.arange(min(x), max(x)+1, 2.0))
    plt.title("File:"+str(numb) + " ;" + angle+ '
Individual peaks fit')
    plt.plot(x, residual, color = 'grey', label = "
residual")
    plt.axhline(y = 0, xmin= x_start, xmax= x_end, color
= 'black') #zero reference line
    plt.xlim([xleft, xright])
    plt.grid()
    plt.legend(title="\n".join(m_singlet_fit_info), loc='
upper left')
    plt.show()

 #Printing results
***********************************************
    print(f"Results for data file SW_Shiva{numb}.txt:")

    for key, value in zip(m_singlet.parameters, m_singlet
.values):
        print(f"{key} = {value}")
```

```
    #*****************Saving results in CSV file
   ******************************************

    # Extract the values of center parameters from m_singlet.
   values
        center_values = [m_singlet.values[f'center_{i}'] for
   i in range(1, 7)]

    # Construct the row dictionary
        row_dict = {
            'file': numb,
            'center_1': center_values[0],
            'center_2': center_values[1],
            'center_3': center_values[2],
            'center_4': center_values[3],
            'center_5': center_values[4],
            'center_6': center_values[5],
            'Comment': row_dict['Comment']  # Include the '
   Comment' key as well
        }
        if np.any(np.abs(residual) > 0.1):
            row_dict['Comment'] = 'Check!!!'
        # Write row to CSV file
        writer.writerow(row_dict)
```

Listing S1: Code for deconvolution of NMR spectra

```
;zg
;avance-version (12/01/11)
;1D sequence
;
;$CLASS=HighRes
;$DIM=1D
;$TYPE=
;$SUBTYPE=
;$COMMENT=


#include <Avance.incl>


"acqt0=-p1*2/3.1416"


1 ze
2 30m do:f2
```

```
  d1
  (5u pl1):f1
  5u pl22:f2
  p1 ph1
  0.5u cpds1:f2
  go=2 ph31
  1m do:f2
  30m mc #0 to 2 F0(zd)
exit


ph1=0 2 2 0 1 3 3 1
ph31=0 2 2 0 1 3 3 1
```

Listing S2: dpdec.swi pulse program used for data collection

3 Wide Scan Spectrum

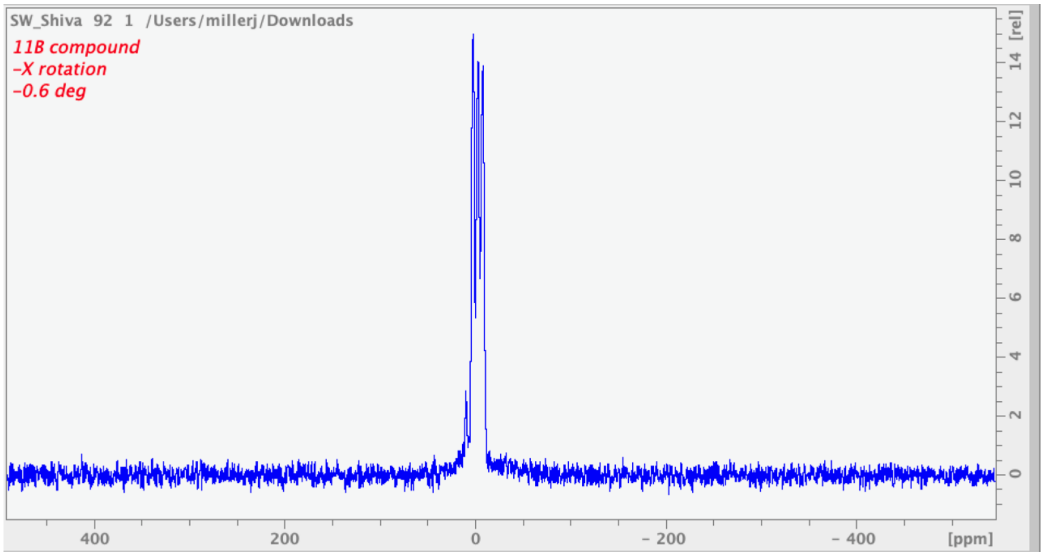

Figure S12: ^{11}B NMR spectrum acquired for (-,-)-8-HQ(ipc)$_2$B sample about the -x^T mounting at a $-0.6°$ rotation angle. The spectrum was examined over a range of ±519 ppm/100 kHz for satellite transitions but none were observed.

4 Orientation of PAF(Q) to Crystal Axis Frame

The Euler angles between the principal axis frame of quadrupolar tensor (PAF(Q)) and crystal axis frame were calculated using the relationship

$$R(\zeta, \lambda, \nu) = R^{-1}(\alpha, \beta, \gamma) R(\alpha_{QG}, \beta_{QG}, \gamma_{QG})$$

where the Euler triplet (α, β, γ) relates crystal axis frame with goniometer frame and was found using x-ray diffraction. The Euler triplet $(\alpha_{QG}, \beta_{QG}, \gamma_{QG})$ gives the orientation of PAF(Q) to goniometer frame.

Compound	Nucleus	α_{QG}	β_{QG}	γ_{QG}	ζ	λ	ν
(+,+)-8-HQ(ipc)$_2$B	1	85	37	335	-53	163	90
	2	319	29	62	81	152	-88
(-,-)-8-HQ(ipc)$_2$B	1	250	60	127	-55	44	-9
	2	58	72	309	24	102	31

Table S1: Experimental Euler angles relating the PAF(Q) to goniometer and crystal axis frame for magnetically equivalent ^{11}B nuclei in (+,+)-8-HQ(ipc)$_2$B and (-,-)-8-HQ(ipc)$_2$B.

References

[1] Klaus Eichele. TRAFO — Coordinate Transformations, 2021.

[2] Ginifab online Protractor.

[3] F. Izumi and K. Momma. VESTA 3 for three-dimensional visualization of crystal, volumetric and morphology data. *Journal of Applied Crystallography*, 44(6):1272–1276, December 2011. Publisher: International Union of Crystallography.
